# Recent Advances in Electrochemical Monitoring of Chromium

**DOI:** 10.3390/s20185153

**Published:** 2020-09-09

**Authors:** Nazha Hilali, Hasna Mohammadi, Aziz Amine, Nadia Zine, Abdelhamid Errachid

**Affiliations:** 1 Laboratory of Process Engineering & Environment, Faculty of Sciences and Techniques, Hassan II University of Casablanca, Mohammedia B.P.146, Morocco; hilalinazha1@gmail.com (N.H.); hasna2001fr@yahoo.fr (H.M.); 2 Institute of Analytical Sciences, University of Claude Bernard Lyon-1, UMR 5280, CNRS, 5 Street of Doua, F-69100 Villeurbanne, France; nadia.zine@univ-lyon1.fr (N.Z.); abdelhamid.errachid-el-salhi@univ-lyon1.fr (A.E.)

**Keywords:** hexavalent chromium, trivalent chromium, electrochemical sensor, electrochemical biosensor, speciation

## Abstract

The extensive use of chromium by several industries conducts to the discharge of an immense quantity of its various forms in the environment which affects drastically the ecological and biological lives especially in the case of hexavalent chromium. Electrochemical sensors and biosensors are useful devices for chromium determination. In the last five years, several sensors based on the modification of electrode surface by different nanomaterials (fluorine tin oxide, titanium dioxide, carbon nanomaterials, metallic nanoparticles and nanocomposite) and biosensors with different biorecognition elements (microbial fuel cell, bacteria, enzyme, DNA) were employed for chromium monitoring. Herein, recent advances related to the use of electrochemical approaches for measurement of trivalent and hexavalent chromium from 2015 to 2020 are reported. A discussion of both chromium species detections and speciation studies is provided.

## 1. Introduction

Chromium is a metallic element, that has been extensively used in various industries, such as those of steel, coating, manufacturing of alloys, tannery of hides, galvanoplasty, treatment of wood and the dying industry [1]. As a consequence, an enormous quantity of different chromium compounds can be discharged in the environment, which may adversely affect the biological and ecological lives.

Different oxidation states of chromium can exist, from Cr (0) to Cr (VI) as the most oxidized form. However, only the oxidation states (III) and (VI) are usually present in the environment owing to their stability [2]. Trivalent chromium (Cr(III)) is essential for the human body, with a quantity of 50–200 µg per day [2]. On the contrary, hexavalent chromium (Cr(VI)) is known by its toxicity, it can penetrate easily inside the cell and damage it [3].

Both short and long exposure of hexavalent Chromium may cause various diseases like ulceration, contact dermatitis, chronic bronchitis, gastrointestinal hepatic, emphysema and pneumonia, hemorrhage, liver and kidney damage and cancer [2,4,5]. It can affect a lot of body organs that may lead to death. The value of 50 µg·L^−1^ was cited by the World Health Organization (WHO) as the provisional guideline value for Cr (VI) in groundwater [6] while 100 µg·L^−1^ was regulated by the Environmental Protection Agency (EPA) for total chromium in drinking water [7]. 

Keeping in mind that the toxicity of chromium depends on its oxidation state, especially its hexavalent form. Accordingly, the development of a method for specific determination of hexavalent chromium is highly desired. Indeed, mercury electrodes have been widely used for chromium speciation [8,9,10]. Some reviews paper about different nanomaterials adsorbent bearing in mind their importance for chromium’s speciation has been published in 2018 [2,11]. Besides, many analytical techniques for trivalent and/or hexavalent chromium measurements have been reported in the literature [12]. The speciation of trivalent and hexavalent chromium was carried out through the development of a rhodamine-based fluorescent agent with the utilization of a fluorimetric method [13]. A coupling between graphite furnace atomic absorption spectrometry and the solid-phase microextraction process was accomplished for chromium speciation in water [14]. A coupling between HPLC analysis and UV technique along with the use of solid-phase extraction were utilized for the separation and speciation of Cr(III) and Cr(VI) in aqueous medium [15]. Suppressed conductivity and its combination with ion chromatography were employed for Cr(VI) measurement [16,17]. Solid-phase spectrophotometry technique was proposed for the preconcentration of chromium on a solid matrix, aided by 4-(2-benzothiazolylazo)2,20-biphenyldiol as a complexing agent followed by the measurement of the absorbance of the formed complex in the solid phase. In this technique, the system was used for total chromium determination, through the measurement of Cr(III) presents in the sample and the reduced Cr(VI) to Cr(III). Moreover, the determination of Cr(VI) was possible via the calculation of the difference between the total Cr and the Cr(III) content [18]. Flame atomic absorption spectrometry was utilized for the determination of chromium(III) in water samples at trace levels (µg·L^−1^) after its preconcentration by graphene [19]. Colorimetric method was also utilized for Cr(VI) determination, based on the functionalization of silver nanoparticles by polyvinylpyrrolidone [20]. 

The standard or conventional methods, known for chromium determination, are atomic absorption spectroscopy, ion chromatography and the colorimetric method with the use of diphenylcarbazide. 

Indeed, the ion chromatography allows the separation of polar molecules and ions based on their affinity to the ion exchanger. While the atomic absorption spectroscopy is based upon the quantitative determination of different chemical elements at their atomic form in the gaseous state via measuring their absorption of optical radiation. The use of these techniques presents several drawbacks such as expensive cost, the need for a qualified person, the complexity of materials and the different steps required for the analysis.

The colorimetric method for the determination of Cr(VI) is based on its complexation with diphenylcarbazide in an acidic medium for 10 min, then, the produced red-violet complex can be measured by spectroscopy at 540 nm [21]. The use of the colorimetric technique is limited to non-turbid and non-colored samples. 

Most of the existing analytical techniques present their lack of flexibility and do not allow simultaneous analysis of the compounds. Hence the interest of developing electrochemical techniques that present a good alternative thanks to their low cost, simplicity, sensitivity and selectivity. 

**Figure 1:**
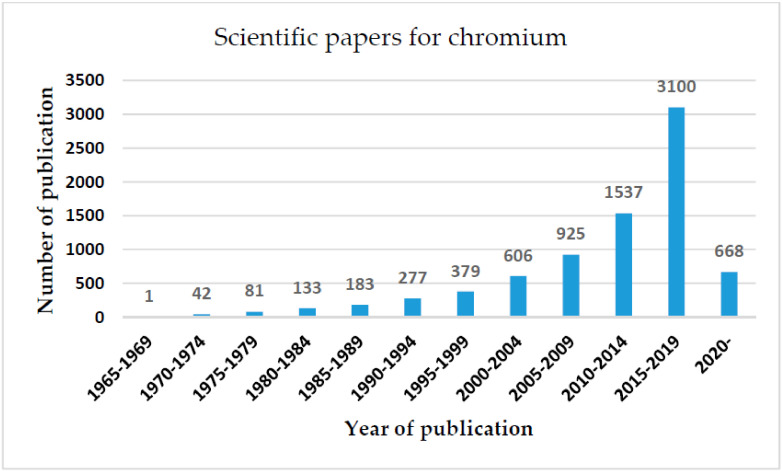
Number of publications in the field of electrochemical detection of chromium in the period between 1965 and 2020 (www.scopus.com, analyzed by 5 years) (Consulted 20-07-2020) (Keywords: (Chromium) and (electrochemical) and (detection or determination or speciation)).

The interest in sensing traces of chromium compounds, in different real samples, has been substantially increased in recent decades. Referring to the data furnished by the SCOPUS database, an important increase in the number of scientific research studies has been devoted to chromium sensing in the last decade. The increasing number of scientific published papers in this field exhibits the importance and the attentiveness given by researchers to the determination of chromium. As stated in Scopus, there is a noteworthy increase in the published scientific papers on electrochemical methods for the determination of chromium species. This increase may owe to the numerous advantages of the electrochemical approaches against the conventional ones (Figure 1)**.**

A review of the recent advances in electrochemical detection of toxic Cr(VI) was published in 2015, in which, different detection strategies and various electrode materials based on mercury, bismuth, carbon and gold for electrochemical detection of Cr(VI) were discussed [3]. Another review paper has been published in 2017, it has outlined different conventional analytical techniques with a particular focus on electrochemical biosensors for chromium detection in potable water [22].

In light of all that has been discussed before, this review aimed to present and discuss the recent advances related to the electrochemical sensing and biosensing of chromium species by direct and indirect strategies conducted in the past 5 years (since 2015) in various real samples. Indeed, the number of papers published since 2015 is equivalent to the total number of papers published before 2015 and thus this review appears timely to update our knowledge on current electrochemical detection of chromium.

## 2. Electrochemical Sensors for Hexavalent Chromium Determination

An electrochemical sensor is a device that transforms the electrochemical interaction between the analyte and the electrode into an analytically useful signal. The two-basic component of a sensor is the sensing material or the receptor, which monitors and detects molecular changes and the transducer which transform the changes observed by the receptor into measurable signals.

**Figure 2:**
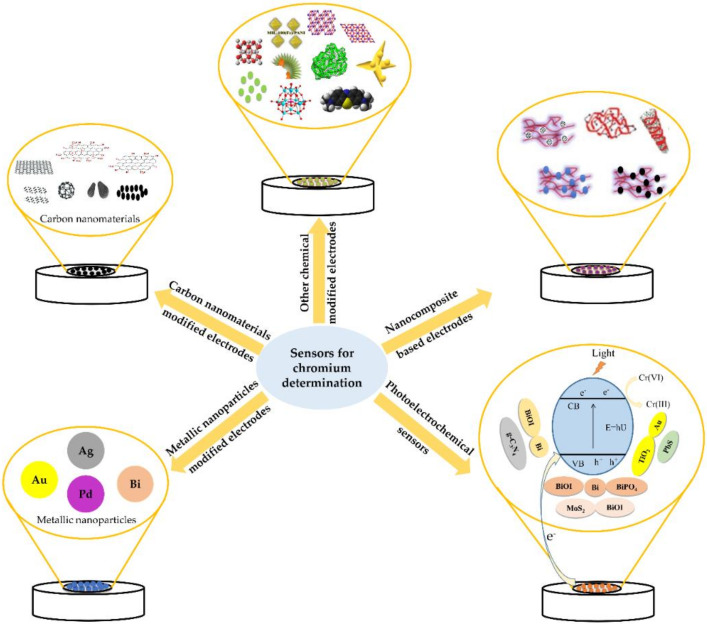
Scheme of different utilized nanomaterials for chromium determination.

Nowadays, nanotechnology has been becoming more and more important. Different nanomaterials actively modified electrodes have come up with enormous advantages, for instance, the increase of the surface-to-volume ratio, low cost, high porosity, ease of modification,… [23,24,25,26,27]. Also, these modifications allow to avoid the overpotential [28], interference problems [28], surface fouling [29] and provides long lifetime of 3 months [30]. Several combinations between different nanomaterials and the use of chemicals as catalyst compounds to modify electrodes for chromium species monitoring in different real samples took place (Figure 2).

### 2.1. Carbon Nanomaterials Based-Electrodes

Carbon nanomaterials exhibit several advantages. In fact, Wei Jin et al. have spotted its advantages in their review [3]. Such as the ease of preparation, renewability, mechanical and chemical stability, also a high electrical conductivity. Besides, the ability of the functionalization of their surface by different functions. Also, they are broadly used in the modification of different electrodes to enhance the sensitivity and obtain a low detection limit [3]. The use of carbon nanomaterials as sensing elements during the last 5 years still taking place (Table 1).

Printed carbon nanotubes (CNTs) onto the different electrodes (screen printed electrode, flexible paper electrode and onto fluorine-doped tin oxide glass) was carried out to fabricate specific amperometric sensor for Cr(VI) measurement. The use of CNTs allows the increase of the current response, resulting in the determination of Cr(VI) at trace levels at a concentration as low as 5 µg·L^−1^ [31]. Similarly, the modification of glassy carbon electrode (GCE) by carboxylated single-walled carbon nanotubes and pyridinium functionalized sol-gel thin film was carried out successfully by Samuel M. Rosolina and coworkers for Cr(VI) monitoring by square wave voltammetry (SWV) [32]. The accumulation of chromate took place on the protonated pyridinium, followed by chromium reduction, resulting in its stripping from the electrode surface and subsequent detection. The addition of SWCNTs reduced the limit of detection from 4.6 µg·L^−1^ [33] to 0.8 µg·L^−1^ [32].

Besides carbon nanotubes, graphene nanomaterial can also be utilized for Cr(VI) detection. Chenglun Liu et al. [34] have successfully modified the GCE by graphene as a sensing device for Cr(VI) measurement. According to the authors [34], the -OH and –COOH group of graphene can easily became cations (-OH_2_^+^ and -COOH_2_^+^), that attract chromium anions (HCrO_4_^−^, CrO_4_^2−^ and Cr_2_O_7_^2−^) in acidic solution through electrostatic interaction that increase the Cr(VI) concentration on the electrode surface. The adsorbed Cr(VI) was reduced to Cr(III) with functional groups as C=O and C-OH, which were oxidized in turn to -COOH. Thus, the reduction reaction of Cr(VI) could be observed electrochemically. The addition of graphene accelerates electron transfer and diminishes the transfer resistance by about nine times in comparison with bare GCE allowing the detection of Cr(VI) at 7.8 µg·L^−1^ with a satisfactory recovery of 99–102% [34]. In another approach, an electrochemiluminescence (ECL) sensor based on the quenching of the cathodic ECL signal of Cr(VI) on the graphene quantum dots and peroxodisulfate was reported. The increase of graphene quantum dots concentration leads to the decrease of the relative standard deviation of the electrochemiluminescence intensity in parallel to the increase of the signal/noise ratio. The developed sensor showed great importance for detecting Cr(VI) concentration as low as 1.04 µg·L^−1^ with high accuracy [35]. To achieve low detection limits for Cr(VI) and high sensitivities, carbon black can be used for electrodes modification owing to its several advantages, such as large surface area, environmentally friendly, high chemical and mechanical stability, high electrical conductivity and very low cost [36,37,38]. Besides, the utilization of such nanomaterial for sensor modification leads to a great enhancement in sensitivity. Yali Wang et al. [39] have developed a novel amperometric sensor for Cr(VI) monitoring in lake water. Carbon black was introduced into the fabrication of the working electrode allowing the enhancement of the electroconductivity of the synthesized polyoxometalate based isomorphous crystalline materials destined for Cr(VI) analysis. The developed sensor exhibits an extremely fast response time (1 s), remarkably low limit of detection 1.35 µg·L^−1^ and a good sensitivity of 3.43 μA·µM^−1^·cm^−2^. Similarly, carbon black modified GCE was fabricated for Cr(VI) measurement [40]. The enhancement of the sensing efficiency took place owing to the sp2-bonded carbon black, which possesses a great quantity of mesopores as the charge transport channels in the interior surface. The developed sensor displays a good sensitivity of 2.79 μA·μM^−1^·cm^−2^ and reached detection limit of 0.52 µg·L^−1^ [40]. However, the dispersion of polyoxometalate-carbon black in nafion solution [39] decreases the accessibility of the carbon black mesopores. It is supposed that, most likely, a repulsion between nafion and chromate anion took place resulting in low sensitivity and high detection limit compared to the carbon black modified GCE [40].

Table 1 shows a comparison between the analytical performance of the cited strategies based on the utilization of different carbon nanomaterials for Cr(VI) detection in several real samples. Their incorporation as sensing nanomaterials leads to an enhancement of the sensitivity, selectivity, good recoveries and allows the determination of Cr(VI) at trace levels (µg·L^−1^) in comparison with the unmodified electrodes.

### 2.2. Metallic Nanoparticles-Based Electrodes

Over the last years, considerable attention has been focused on the evolution of nanoscience and nanotechnology. Among the nanomaterials, metal nanoparticles have taken a broad interest as nanosensors, biological labeling and catalyst. This enormous use of those nanoparticles for the elaboration of sensors was explained by their large surface-to-volume ratio, surface reaction activity, high electrical conductivity and great catalytic ability [41].

Several metallic nanoparticles have been used for chromium monitoring. Sixing Xu et al. [42] have proposed a novel sensor based on the modification of mesoporous carbon electrode by bismuth film for Cr(VI) sensing at 0.05 µg·L^−1^ concentration. The mechanism of the detection was based on the preconcentration and adsorption of the formed chelate between diethylene triamine pentacetate acid (DTPA) and Cr(III) stemmed from reduced Cr(VI) on bismuth. The fabricated sensor showed the ability of the detection of Cr(VI) with a low background noise due to the decoration of the carbon frame by bismuth electrodeposited. In another work, electroplating of bismuth onto the glassy carbon electrode with a reversibly deposited zinc mediator in the presence of DTPA has been carried out successfully by Katarzyna Tyszczuk-Rotko and coworkers [43]. The use of zinc as a mediator enhanced considerably the intensity of the analytical signal of Cr(VI), 1 µA vs. 3.3 µA for bismuth film electrode prepared without and with zinc mediator respectively leading to the detection of an extremely low concentration of 3.01 × 10^−6^ µg·L^−1^. The developed sensor was applied for Cr(VI) monitoring in river water with a recovery of 95–97%.

Apart from bismuth, the incorporation of silver as a sensing material for the determination of chromium has also gained great interest, owing to its catalytic effect. Generally, metal nanoparticles have excellent catalytic and conductive properties which make them more suitable to act as electronic wires to improve the transfer of electrons between redox centers on the surface of electrodes and also as catalysts to promote electrochemical reactions [44]. Zorica Stojanović et al. [45] have fabricated a sensor based on the in-situ electroplating of GCE by a silver film for Cr(VI) sensing at µg·L^−1^ level by differential pulse anodic stripping voltammetry. The electroplating of silver produced signals of high quality, enhanced the sensitivity 4 times than the bare electrode and exhibits the detection of Cr(VI) at 5.2 µg·L^−1^ level. Also, the fabricated sensor allows the determination of Cr(VI) with a satisfactory recovery of 97–105% in tap water. In another study effectuated by Shahbakhsh et al. [46], graphite paste electrode was modified by decorated silver nanoparticles on two dimensional (2D) –biphenol biphenoquinone (BP/BPQ) nanoribbons for indirect measurement of Cr(VI) through the decrease in current intensity of BP/BPQ redox system. As indicated in the literature [46], BP should be a two electron and two proton process. Also, HCrO_4_^−^ is the dominant specie at pH 2 and lower concentration of Cr(VI). For that, the redox reaction between BP/BPQ and Cr(VI)/Cr(III) is: 3BP + 2HCrO_4_^−^ + 8H^+^ → 3BPQ + 2Cr^3+^ + 8H_2_O. The AgNPs enhance the current intensity and increase the reversibility of the fabricated sensor. Moreover, electron transfer became faster. The developed sensor allows the detection of Cr(VI) in different water samples with a good recovery range of 97–103% and a detection limit of 1.04 × 10^−4^ µg·L^−1^ [46].

**Figure 3:**
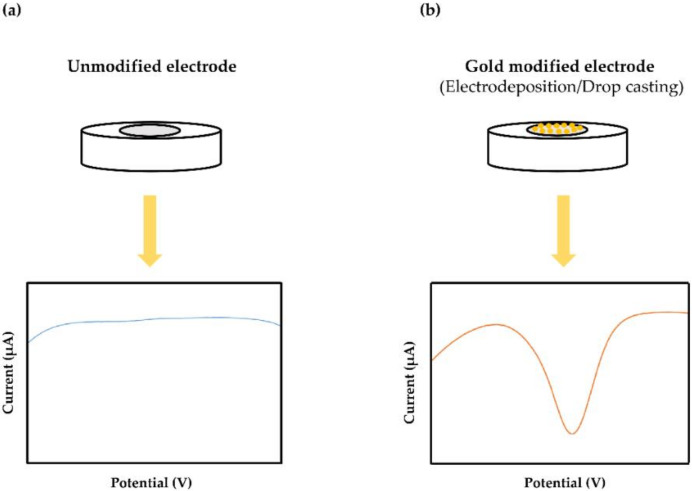
Typical response of Cr(VI) at: (**a**) unmodified; (**b**) AuNPs modified electrode using electrodeposition or drop casting strategy.

Over the last decade, gold nanomaterials (AuNPs) have intended great interest as an incorporated sensing material for the elaboration of sensors for chromium monitoring using several modification strategies. It was also used as an efficient catalyst for the reduction of Cr(VI) (Figure 3).

Detection of Cr(VI) by gold nanoparticles has multiple strategies. Amongst, electrodeposition and drop-casting are the most common. Indeed, Salamatu Aliyu Tukur et al. [47] have modified the screen-printed electrode by gold nanoparticles for Cr(VI) measurement at pg level in water samples. According to the authors, the detection method was based on the reduction of hexavalent chromium to chromium metal onto the electrode surface, followed by the detection of chromium via its oxidation according to the following equations:

$$\begin{array}{l} {\mathrm{Deposition}\;\mathrm{step}:\;\mathrm{HCrO}}_{4^{-}}{+\;H}^{+}↔H_{2}{\mathrm{CrO}}_{4}\overset{{+e}^{-}}{\to }{\mathrm{CrO}}_{3^{-}}{+\;H}_{2}O\overset{{6H}^{+}{,\;3e}^{-}}{\to }{\mathrm{Cr}}^{3+}{+\;3H}_{2}O\overset{{3e}^{-}}{\to }\mathrm{Cr}\left(m\right)\\ \mathrm{Stripping}\;\mathrm{step}:\;\mathrm{Cr}\left(m\right){\;+\;6H}^{+}{+\;3/2O}_{2}\overset{{-3e}^{-}}{\to }{\mathrm{Cr}}^{3+}{+\;3H}_{2}O \end{array}$$

This is one of the rare methods for chromium determination and it allows the determination of Cr(VI) at a concentration as low as 1.6 × 10^−3^ µg·L^−1^. Owing to their catalytic effect [48], AuNPs significantly enhanced the peak current by 27 folds along with the enhancement of the microscopic surface area and the rate of electron transfer. The fabricated sensor exhibits numerous advantages including high sensitivity, high selectivity, low sample volumes, ease of fabrication. In another study, the self-assembly process was utilized by Santhy Wyantuti et al. [49] for the activation of the GCE to maximize its covering by AuNPs. Hydrogen group can be substituted with amine group during the self-assembly. More AuNPs can be attached to the electrode surface thanks to the nature of the amine group. Cyclic voltammetry was conducted for the detection of Cr(VI) in a wide linear range 0.05–0.25 µg·L^−1^ with a limit of detection at ng·L^−1^ level (2.38 ng·L^−1^). Later, an electrochemical miniaturized portable system based on a screen-printed carbon electrode modified with AuNPs has been designed by Jiawei Tu et al. [50] for Cr(VI) measurement in river water with a good recovery range of 90–106%. The electrochemical system included an analyzer, a detection module combined with the working electrode and a laptop/smartphone, was well designed for in-situ applications and showed a detection limit of 5.4 μg·L^−1^. Also, a comparison between gold nanomaterials form has been carried out recently between gold nanostar (AuNSs) and a spherically shaped gold nanoparticles modified screen-printed electrode for Cr(VI) measurements in an acidic medium by Susom Dutta and coworkers [51]. The AuNSs provided the highest current response to Cr(VI) in comparison with AuNPs with a detection limit of 3.5 µg·L^−1^. The prepared sensor was utilized for the determination of Cr(VI) in contaminated groundwater with an average recovery of 96%. Moreover, Huy Du Nguyen et al. [52] reported a new voltametric sensor built on the modification of a platinum rotating disk electrode by gold nano-flakes and 4-pyridine-ethanethiol through electrodeposition approach. The developed sensor showed excellent properties for Cr(VI) sensing in Coastal water with enhanced sensitivity and reproducibility and a very low detection limit (0.001 µg·L^−1^).

Most of the studies described above have shown that an adequate determination of hexavalent chromium requires an acidic medium. In general, for low concentrations of Cr(VI), the main reactions are (1) and (2) [53,54]:

H_2_CrO_4_ ⇆ HCrO_4_^−^ + H^+^ (1)

HCrO_4_^−^ ⇆ CrO_4_^2−^ + H^+^ (2)

At pH lower than 1, the hexavalent chromium exists under the form of H_2_CrO_4_ as salts of chromic acid, it also exists as hydrogen chromate ion (HCrO_4_^−^) at pH ranges from 1 to 6 and as chromate ion (CrO_4_^2−^) when pH value exceeds 6. The formation of the dimer of HCrO_4_^−^ under the form of the dichromate ion (Cr_2_O_7_^2−^) took place when the concentration of hexavalent chromium exceeds 1 g·L^−1^ as indicated in equation (3) [53,54]:

2HCrO_4_^−^ ⇆Cr_2_O_7_^2−^ + H_2_O (3)

Alongside the decrease of pH, the concentration of HCrO_4_^−^ and H_2_CrO_4_ increases (Equations (1) and (2)). In the acidic medium, the HCrO_4_^−^ is the electroactive species of chromium, which explains the extensive use of the acidic medium for the determination of Cr(VI) [50].

Chloride ions affect adversely the determination of Cr(VI) at sensors based on gold nanoparticles as reported recently in our previous work, in which we investigate the effect of chloride ions at AuNPs electrodeposited and drop casted onto carbon paste electrodes [55]. Both modified gold electrodes showed limitations for the detection of Cr(VI) in the presence of chloride ions owing to the possibility of forming the complex of CrO_3_Cl^−^. The current intensity of Cr(VI) decreased drastically in tap water due to the presence of chloride ions. In the same work, an unmodified carbon paste electrode (CPE) has been tested for Cr(VI) monitoring in the presence and absence of diphenylcarbazide. The use of unmodified CPE in the presence of diphenylcarbazide allowed the measurement of Cr(VI) even in the existence of chloride ions thanks to the high affinity of Cr(VI) to form a complex with diphenylcarbazide compared to that of chloride [55].

Different functionalization by metallic nanoparticles has been carried out for Cr(VI) determination. The analytical performances of the cited modified metallic nanoparticles electrodes are summarized in Table 2. This type of nanomaterials offers important improvement to Cr(VI) detection, such as enhancement of the selectivity, reproducibility, increasing linearity ranges and improving detection limits.

### 2.3. Nanocomposite Based-Electrodes

Unique and fascinating characteristics of nanocomposite materials have attracted substantial attention over the last years. Several combinations of nanomaterials have been investigated for improving the performance of the sensors [27,56,57]. The incorporation of nanocomposites as sensing material affords various advantages including, high selectivity, high surface area and fast electron-transfer rate of the electroactive species to electrode surface [58,59,60,61,62]. Moreover, those nanocomposites provide high electrical conductivity thanks to the synergistic effect which is rarely explained in the literature. In fact, in case of bimetallic nanoparticles, higher selectivity and activity are offered when compared with the monometallic ones that might be the consequence of electron charge transfer between the used metal nanoparticles. In case of non-alloy systems, it may due to the formation boundaries between two metal phases [63,64,65,66].

Trisna K. Sari et al. [67] have developed a composite working electrode from graphite and styrene-acrylonitrile copolymer for Cr(VI) detection in different water samples with satisfactory recoveries of 97–101%. The mechanism is based on the reduction of Cr(VI) to Cr(III) on the electrode surface during the preconcentration step followed by an anodic stripping. The as developed sensor showed high mechanical rigid, ease of regeneration, high sensitivity and superior performance than glassy carbon electrode thanks to the strong interaction between the nitrile end group of the prepared copolymer and Cr(III), with a detection limit of 4.5 µg·L^−1^.

The use of nanocomposites based on the combination of ion imprinted polymers (IIP) and carbon nanomaterials for Cr(VI) detection has also a share. Songjun Li et al. [68] constructed a new sensor based on ion-imprinted chitosan-graphene nanocomposites modified gold electrode dedicated to the detection of Cr(VI) with recoveries of 99–104% and 96–97% in tap and river water respectively. The designed sensor showed high selectivity owing to the created cavities in the IIP and the metal-ligand chemistry, excellent stability, good repeatability and low limit of detection of 3.33 × 10^−2^ µg·L^−1^.

Very recently, a modification of glassy carbon electrode by graphene carbon nitride decorated silver molybdate immobilized with nafion was used as a platform for sensitive determination of Cr(VI) [58]. Moreover, the incorporated nanocomposite possessed a high active surface area, enhanced sensitivity, selectivity, repeatability and reproducibility. Besides, the developed system allows the detection of Cr(VI) at 8.32 × 10^−2^ µg·L^−1^ level.

Weidong Kang et al. [59] reported a new amperometric sensor based on NiFE bimetallic nanoparticles for sensitive detection of Cr(VI) in groundwater with good recovery of 98–104%. The NiFE possessed multiple oxidation states which make the surface very active. The prepared sensor exhibits good accuracy, high sensitivity, simplicity, a wide linear range 1.3–5112 µg·L^−1^ and a detection limit of 0.52 µg·L^−1^. In another approach, gold nanoparticles were combined with different carbon nanomaterials and/or polymers for the elaboration of nanocomposites intended for Cr(VI) sensing. The 3D NiO/Polyaniline foam is another electrode material that helped in detecting Cr(VI) by using interfacial potential barriers [69]. The mechanism is based on the increase of the potential barrier height of p-p junction after the adsorption of HCrO_4_^-^ on the film of polyaniline leading to an electrochemical current decrease. This new approach exhibits many advantages including an extremely low detection limit of 2.06 × 10^−5^ µg·L^−1^, high sensitivity compared with the majority of cited electrochemical strategies.

Annamalai Senthil Kumar et al. [70] reported a flow injection analysis with a dual electrochemical detector at 0.1 V and 1 V vs. Ag/AgCl for Cr(VI)-reduction and Cr(III)-oxidation respectively. In this work, gold nanoparticles designed carbon nanofibres-chitosan was used as an electrode modified material. The incorporation of AuNPs in the prepared carbon nanofibres-chitosan was the key to the successful activity of the sensor toward Cr(VI) because their absence leads to non-response signal. The developed sensor was employed for the detection of Cr(VI) in industrial wastewater with a satisfactory recovery of 98–103% and 0.32 µg·L^−1^ as the lowest concentration detected. In their work, Trisna K. SARI et al. [60] developed an amperometric sensor based on the modification of glassy carbon electrode by graphene-AuNPs for Cr(VI) sensing in river water with good recovery of 101–104%. The developed sensor exhibits the highest electrocatalytic activity for the Cr(VI) reduction, also high stability, high sensitivity and low detection limit of 0.52 µg·L^−1^. Similarly, modification of GCE by reduced graphene oxide and AuNPs through electrodeposition process was carried out by Ye Liu et al. for the determination of Cr(VI) in lake and river waters with a recovery of 93–114% [71]. The growth and nucleation of metal nanoparticles on the rGO surface took place owing to the residual oxygen on its surface. Furthermore, the aggregation of metallic nanoparticles can be prevented due to the large specific surface area of rGO. The elaborated sensor exhibits numerous advantages, including excellent stability, high sensitivity, good selectivity and low detection limit of 2.392 µg·L^−1^. In a similar context, Carmel B. Breslin and coworkers [61] have proposed a novel amperometric sensor based on MWCNTs decorated AuNPs on gold electrode as a platform for Cr(VI) measurement. The prepared composite material offered several advantages, among them the high conductivity provided by MWCNTs and the high surface area which facilitates the AuNPs nucleation. The elaborated sensor showed a quite good detection of Cr(VI) at 37.44 µg·L^−1^ level, with good selectivity.

Another strategy based on the precipitation of a thin layer of the copolymer poly(aniline-co-o-toluidine)/graphene oxide followed by the electrodeposition of AuNPs on the surface of gold electrode was effectuated by the research team of Mahmoud A. Hussein [62]. graphene oxide increases the porosity of the nanocomposite and AuNPs provide high electron transfer. This sensor allows the detection of Cr(VI) in tap water with a recovery of 94–117% with a concentration as low as 1.12 µg·L^−1^. Moreover, it displayed a good selectivity, sensitivity and stability for measurement. In another study, a nanocomposite of polyaniline and graphene quantum dots was utilized for the modification of a screen-printed carbon electrode for Cr(VI) detection [72]. The detection was realized by a stop-flow analysis coupled with a linear sweep cathodic voltammetry technique. The developed sensor showed high current response thanks to the conductivity provided by polyaniline and the properties cited above of GQD, a fast response (less than 1 min), a detection limit of 97 µg·L^−1^ and satisfactory recovery of 80–106% for Cr(VI) measurement in mineral water [72]. Recently, a GCE modified with graphene and pyridine functionalized gold nanoparticles was successfully exploited by Yiwei Xu and coworkers [73] to detect hexavalent chromium by differential pulse adsorptive stripping voltammetry at a concentration as low as 1.16 μg·L^−1^. Large surface area, excellent electric conductivity and strong complexation ability are provided by graphene, AuNPs and pyridine group respectively. The as-prepared electrodes were applied to determine Cr(VI) in wastewater samples with a satisfactory recovery of 97–105%.

In another work, Fei Li et al. developed a new voltametric sensor for Cr(VI) monitoring in coastal water with recovery in the ambit of 96–105% [74]. Modification of GCE was effectuated by cubical nano-titanium carbide (Nano-Tic) used as the supporter for AuNPs loading. The developed sensor provided an excellent response toward Cr(VI) with a high sensitivity thanks to the synergistic effects of Nano-TiC and AuNPs. It also provides a wide linear range from 5.2 µg·L^−1^ to 1040 µg·L^−1^ with a detection limit of 2.08 µg·L^−1^. Ag-doped TiO_2_ nanoparticles is another interface nanomaterial used for Cr(VI) monitoring in various water samples by Amperometry [75]. In this, the Ag-doped TiO_2_ was employed to modify the GCE. Also, this combination has enhanced the selectivity and exhibits a detection as low as 0.52 µg·L^−1^. Besides, the prepared materials showed better photocatalytic activity in sunlight compared to UV light.

Honggui Wang et al. [76] have published a novel electrochemical sensor based on the modification of GCE by Fe_3_O_4_/MoS_2_ for Cr(VI) monitoring. The electrochemical activity and the current intensity were higher for Fe_3_O_4_/MoS_2_ /GCE compared with bare GCE, along with pure Fe_3_O_4_ or MoS_2_ modified electrode. Under the optimized conditions, the fabricated sensor showed a good electrocatalytic performance for Cr(VI) in a wide linear range from 52 µg·L^−1^ to 136760 µg·L^−1^, with a detection limit of 26 µg·L^−1^.

In another approach, the bimetallic AuPd nanoparticles and reduced graphene oxide were also utilized in the construction of a composite sensor intended for Cr(VI) measurement [77]. Owing to the numerous advantages among them facile preparation process (One-step electrochemical approach), high selectivity, sensitivity and stability, the prepared sensor showed great potential for the Cr(VI) monitoring with recovery more than 95% in lake and river waters and 0.676 µg·L^−1^ as the lowest detected concentration.

It is worth pointing out that the incorporation of bimetallic AuPdNPs as sensing materials for the elaboration of nanocomposite sensors showed a higher performance for the determination of Cr(VI) than the incorporation of one metal with a carbon nanomaterial. AuPdNPs/ErGO/GCE provided the lowest limit of detection in comparison with the AuNPs-carbon nanomaterial composite sensors (Table 3). In a similar concept, Gregory Kia Liang Goh et al. [66] have developed a bimetallic (gold-palladium) nanoparticles decorated indium tin oxide for Cr(VI) monitoring. The developed sensor allowed the determination of Cr(VI) even in the presence of several ions among them Cl^-^ without any interferences. The developed sensor exhibits the lowest limit of detection (1.56 × 10^−2^ µg·L^−1^) among all composite sensors, which can be explained by the synergistic effect of gold-palladium. Moreover, the IIP-S sensor [68] provided excellent detection limit (3.33 × 10^−2^ µg·L^−1^) of Cr(VI) as it is shown in Table 3. The highest sensitivity may be due to the large surface area given by graphene and to the created cavities in the IIP (strong metal-ligand chemistry)

Compared to the modified carbon electrodes and modified metallic nanoparticles electrodes, the nanocomposite ones (Table 3) provide the highest analytical performance resulting in enhanced catalytic effect, improving selectivity, detection limit and allowing wide linear range of Cr(VI) with good recoveries in different real samples. Such advantages are related to the synergetic effect of the used nanomaterials. Carbon nanomaterials provide high surface area, enhance the porosity of the nanocomposite, in case of nanocomposite with metallic nanoparticles aid in the prevention of their aggregation and the capacity of seizing Cr(VI) by different functional groups in case of functionalization of the nanocomposite. Furthermore, metallic nanoparticles offer large surface area, high conductivity and high electron transfer between the electrode surface and the analyte.

### 2.4. Photoelectrochemical Sensor

The photoelectrochemical sensor is a new type of analytical device. The mechanism is based on the photoelectrochemical properties of materials including titanium dioxide and fluorine tin oxide [78,79,80]. Owing to its remarkable sensitivity and its ease of integration, photoelectrochemical sensors (PEC) are becoming favorable analytical devices.

A nanostructured photoelectrochemical sensing element based on the use of Cr(VI) ion imprinted polymer and formate ion incorporated graphitic carbon nitride has been explored by the Tian Fang group [78] for Cr(VI) measurement in different water samples with good recovery of 89.8–105% at 0.006 µg·L^−1^. The designed sensor exhibits high selectivity and sensitivity towards Cr(VI) owing to the efficient photogenerated electron reduction offered toward Cr(VI).

During the last years, extensive use of BiOI has been carried out for the elaboration of the photoelectrochemical sensor (PEC) owing to its excellent absorption ability of visible light. However, its use is limited by its low energy conversion efficiency [81,82,83]. Recently, Mengying Li et al. [81] developed a PEC sensor designed by composites of BiOI along with Bi/BiOI-X for Cr(VI) monitoring in water samples with a satisfactory recovery of 98–102% at a concentration as low as 15.6 µg·L^−1^. The mechanism of detection was based on the reduction of chromium specie from Cr(VI) to Cr(III) by photogenerated electrons. Then, the consumed electrons lead to the enhancement of photocurrent density. The same research group proposed another PEC sensor for Cr(VI) detection based on bismuth surface plasmon resonance fostered BiPO_4_/BiOI heterostructures [82]. An improvement of the energy conversion efficiency has carried out owing to the benefits of the p-n heterojunction structure of BiPO_4_/BiOI and the effect of Bi metal SPR. They suggested that the designed PEC has excellent selectivity toward Cr(VI), a wide linear range from 26 µg·L^−1^ to 9360 µg·L^−1^ and a low detection limit of 15.6 µg·L^−1^. In another work, Cheng Chen et al. [83] reported the modification of indium tin oxide by MoS_2_/BiOI for Cr(VI) detection under the visible light irradiation. The adequate amount of MoS_2_ provided rapid electron transfer and good electrical conductivity. However, the excess of MoS_2_ affects the ability of the constructed material to absorb visible light. The as prepared sensor allows the determination of Cr(VI) in tap and lake water with excellent recovery of 98–100% at the level of 0.52 µg·L^−1^.

In another approach, the Junwei Di group [80] developed a new PEC sensor modified with PbS quantum dots onto the surface of ITO for measuring Cr(VI) in water samples with a recovery of 91.8–98.2%. The generated photoelectrons by the PbS QDs were captured by Cr(VI) leading to the separation of electron holes and intensification of the generated photocurrent. Such proposed p-type semiconductor offers various advantages such as low cost, short response time, visible-light irradiation, good sensitivity and detection limit of 5.2 × 10^−4^ µg·L^−1^.

Modification of FTO glass by single-crystal rutile titanium dioxide nanorods was effectuated through the hydrothermal method followed by their decoration by AuNPs destined to the direct determination of Cr(VI) in tap and river water with recoveries of 98–99% and 98–101% [79]. The proposed sensor demonstrated a fast reduction of Cr(VI) in 30 min instead of 90 min high sensitivity thanks to the usage of AuNPs which diminish the charge transfer resistance with 0.312 µg·L^−1^ as the lowest concentration could be detected. Similarly, Roozbeh Siavash Moakhar et al. have decorated a screen-printed TiO_2_ with gold nanoparticles for direct Cr(VI) monitoring at a concentration as low as 0.208 µg·L^−1^ [84]. The fabricated sensor provides a high density of reactive sites, an additional electrical surface charge provided by gold nanoparticles, good reliability, accuracy, rapid response, great sensitivity of 11.88 µA·µM^−1^. Also, Au doped TiO_2_ nanoparticles based GCE sensor have been investigated toward Cr(VI) analysis by Ravishankar and coworkers [85]. The elaborated sensor was employed for amperometric detection of Cr(VI) in tap and industrial wastewater with a great recovery ranges from 98% to 100% and a detection limit of 520 µg·L^−1^.

It is worth mentioning that the majority of the cited photoelectrochemical sensors (Table 4) present the aptitude of the determination of Cr(VI) even in the existence of high concentrations of chloride ions thanks to their high sensitivity and selectivity towards Cr(VI) due to the efficient photogenerated electron reduction offered toward Cr(VI).

### 2.5. Other Sensors

In addition to the already mentioned strategies for the determination of Cr(VI), it exists other sensors that will be discussed in this paragraph.

Nowadays, great efforts have been provided to the utilization of polyoxometalates (POMs) as functional catalysts in several applications among them catalysis [86,87,88], energy conversion [89,90,91] electronics and biosensors [92,93,94,95,96,97]. Recently, Zhangang Han et al. [39] have published two papers about the elaboration of POMs based sensors for Cr(VI) detection. Three types of phosphomolybdates {P_4_Mo_6_} cluster based crystalline materials were synthesized with three different central metals (Co, Ni and Cd). The GCE modified by the synthesized material with Co as a central metal provides the highest current response to Cr(VI) with high sensitivity in a range of (52 to 39,312 µg·L^−1^), a detection limit of 2.7 µg·L^−1^ which might be due to the synergistic effect between the phosphomolybdate and central Co metal [39]. Later, the same team [98] has reported an electrochemical sensor based on two reductive POMs based crystalline material for Cr(VI) determination. Compound 1 “(H_2_bpp)_2_[Na_4_Fe(H_2_O)_7_][Fe(P_4_Mo_6_O_31_H_6_)_2_]·2H_2_O” reveals a unique three-dimensional inorganic porous framework while the compound 2 “(H_2_bpp)_6_(bpp)_2_[Fe(P_4_Mo_6_O_31_H_8_)_2_]_2_·13H_2_O” exists discretely in 0D. such difference has a significant effect on the electrochemical performance of the electrocatalysts. Compound1/GCE showed excellent electroanalytic performances for Cr(VI), linear range from 104 to 135,720 µg·L^−1^ and a detection limit of 9.05 µg·L^−1^ [98]. Besides, the use of those crystalline catalyst compounds as sensing materials showed an excellent selectivity toward the analyte, good stability, reproducibility as well as high resistance against coexisting interferons.

Indeed, Enrique Barrado et al. have suggested the modification of the screen-printed electrode with magnetic pol(1-allyl-3-methylimidazolium) chloride for Cr(VI) determination in tap water samples. The detection was carried out by adsorptive cathodic stripping voltammetry without the addition of complexing agent unlike the reported studies [42]. The use of magnetic particles grants the ease of modification of the sensor [99].

Modification of SPE by two compounds of α-Fe_2_O_3_ nanostructures for the investigation of the effect of (001) plane exposure on the sensing performance of Cr(VI) was successfully exploited by Li Fu and coworkers [100]. The α-Fe_2_O_3_ nanostructure with high exposure of the plane (001) facilitates the Cr(VI) adsorption due to its increased density of hydroxyl groups. The developed sensor allows the detection of Cr(VI) from 5 to 3000 μg·L^−1^. Besides, its ability to detect Cr(VI) at 1.17 μg·L^−1^ level with good reproducibility and stability. In another approach, a disposable gold electrode was elaborated by Wan Wang et al. for Cr(VI) measurement in tap, lake water and plastics with a recovery ranges from 99–106%. In this study, the Cr(VI) measurement was carried out in the linear ranges of 1–50 and 50–120 µg·L^−1^ with 0.7 µg·L^−1^ as the lowest detected concentration [101].

A chemiresistive sensor elaborated by the use of 1,4-dithiothreitol (DTT) functionalized AuNPs and reduced graphene oxide (rGO) was conducted for Cr(VI) determination [102]. The mechanism is based on the selective binding between the DTT functionalized AuNPs located in rGO conductive channels (DTT-AuNPs1) and DTT functionalized AuNPs in solution (DTT-AuNPs2) through the formation of disulfides bridges induced by Cr(VI) at acidic condition resulting in the aggregation of DTT-AuNPs2 on the surface of rGO channels which produces a measurable resistance change. The fabricated sensor allows fast real-time Cr(VI) monitoring in approximately 50 s. The lowest concentration of Cr(VI) could be detected was in the order of 4.68 × 10^−2^ µg·L^−1^.

Double polymer membrane modified GCE operated via electropolymerization of poly (3-octylthiophene) and drop-casting of poly(vinyl chloride) for the determination of hydrogen chromate ions (HCrO_4_^−^) by stripping voltammetry [103]. Chromate anions had the ability to be trapped in the PVC membrane during the time of preconcentration step (4 min), then stripped exclusively. The prepared sensor provides a selective and sensitive response in a linear concentration ranges from 1.56 µg·L^−1^ to 67.6 µg·L^−1^ and detect Cr(VI) at 0.642 µg·L^−1^ level.

In another strategy, the determination of Cr(VI) occurred by means of the modification of screen-printed electrode by bismuth vanadate (BiVO_4_). The addition of BiVO_4_ enhances the electrocatalytic activity for Cr(VI) detection attributable to the high surface area of the prepared catalyst and to its high crystallinity. Besides, the prepared sensor displayed great electrocatalytic activity, good stability, recyclability (4 times), wide linear range (0.52–13,754 µg·L^−1^) with the ability to detect Cr(VI) at the level of 0.182 µg·L^−1^ [104]. Nanocomposite catalysts are a versatile and powerful tool used for Cr(VI) reduction. For instance, a composite photocatalyst based on the use of the polyaniline and the metal organic framework MIL-100(Fe), which is composed of Fe-O metal clusters, organic linker and trimesic acid, was prepared and analyzed by Chong-Chen Wang et al. [105]. During the light irradiation, the photogenerated electrons in conduction band of MIL-100(Fe), which possesses lower reduction ability, recombine with the photogenerated holes in valence band of polyaniline which has lower oxidation ability. Thus, the photogenerated electrons in polyaniline lead to high reduction ability. However, high oxidation ability resulted by the photogenerated holes in MIL-100(Fe). It results an enhancement of the photocatalytic performances attributed to the so called Z scheme of the aforementioned mechanism [106]. Moreover, it provides good stability and reusability in water samples. In another strategy, Dong-Hau Kuo et al. [107] proposed the synthesis of nanoflower catalyst (In,Ga)_2_(O,S)_3_ loaded by different percentages of V_2_O_5_ for Cr(VI) reduction. Forty percent of V_2_O_5_ loaded (In,Ga)_2_(O,S)_3_ showed the highest reducing capability in 2 min, higher electrical properties and higher electron transfer rate compared with unloaded nanocomposite catalyst, along with other loaded percentages of V_2_O_5_ (30% and 50%).

Layer by layer technique in conjunction with polyaniline and poly(vinyl sulfonic acid) (PVS) was also proposed for the elaboration of thin films fabricated from non-modified microcrystalline cellulose (MC) or MC modified by phosphate group (PC) which improves the conductivity and the stability of PANI in presence of the metal [108]. The prepared sensor (PVS/PANI(PC)/ITO) in combination with square wave voltammetry showed the ability for Cr(VI) monitoring linearly from 759.2 to 3120 µg·L^−1^. It was also tested for Cr(VI) measurement in tap water with a recovery ranges from 80% to 97%. In the same context, natural polysaccharides prepared from agar and carrageenan were deposited by layer by layer self-assembly process alternating with polyaniline onto the surface of tin-doped indium oxide [1]. The prepared film showed improved electrochemical stability of the polyaniline in acid medium owing to the antioxidant activity of the employed agar and carrageenan. Under the optimized conditions, the constructed sensor showed high performance in the Cr(VI) detection linearly from 129 to 2600 µg·L^−1^. The presented study displayed the advantages of the incorporation of natural polymer thin films for the elaboration of electrochemical sensors for Cr(VI) in comparison with the widely used PVS polymer.

Laminar flow fuel cell (LFFC) is also known as membraneless microfluidic fuel cell. It is based on characteristics of the fluid in low Reynolds number regimes (<2100). The LFFC benefits from a simple architecture that eliminates membrane-related problems among them membrane degradation and water management [109]. Owing to its numerous advantages including, short time of hydraulic retention of about 2 min high energy output and fast response time (few minutes), LFFC has opened up novel opportunities for electrochemical activity detection of microorganisms [110,111]. In fact, Dingding Ye et al. [112] have fabricated a laminar flow fuel cell sensor for Cr(VI) monitoring in industrial wastewater. Under the optimal conditions, the designed LFFC sensor detect Cr(VI) at a concentration as low as 2500 µg·L^−1^ in less than 1 min with good reproducibility.

Metal oxide nanoparticles were also used as an effective sensing material for Cr(VI) monitoring. Amongst, nickel oxide (NiO) nanoparticles which were destined to be coated on fluorine doped tin oxide plate. The developed sensor was able to detect Cr(VI) with linearity from 5 × 10^3^ to 50 × 10^3^ µg·L^−1^. More modifications of the sensor by the incorporation of other sensing nanomaterials are needed for enhancing the electrode response and detecting the lowest concentration of Cr(VI) [113].

Several publications reported the utilization of electrochemical sensors based upon the modification of gold solid electrode for the electrochemical monitoring of Cr(VI) thanks to its wide potential window, long term stability and low background current [114,115,116,117]. As indicated in Table 5, different modified gold electrodes were effectively used to quantify Cr(VI). For that, Lee E. Korshoj and coworkers [114] have proposed an electrochemical ion sensor for highly specific detection of Cr(VI) based upon the interaction between Cr(VI) and methylene blue (MB) immobilized onto the surface of the gold electrode. First, MB was reduced at the electrode’s surface to form leucomethylene blue, which reduced Cr(VI) to Cr(III) and regenerate MB in the process. The elaborated sensor showed high sensitivity, selectivity and the aptitude to detect Cr(VI) at 26 µg·L^−1^ level. In another study, an electrochemical impedimetric sensor based on self-assembly of the azacrown monolayer on the gold electrode in a combination with electrochemical impedance spectroscopy has been fabricated for Cr(VI) sensing at trace levels (0.0014 µg·L^−1^). The strategy of detection was based on the high-affinity and the specific binding of the azacrown monolayer toward HCrO_4_^−^. The developed impedimetric sensor exhibits a high selectivity toward Cr(VI) in the presence of other interfering ions over a linear concentration ranges from 1 to 100 µg·L^−1^ and a great recovery of 99.6 ± 3.3% in river water [115]. Another strategy based on direct redox sensing of different concentrations of Cr(VI) linearly from 50 to 400 µg·L^−1^ has been developed via the coating of the Manganese Oxide Nanochips (Mn_3_O_4_) onto the gold electrode’s surface [116]. The use of Mn_3_O_4_ holds various advantages including ease of synthesis, low cost, high electrochemical stability, the ability to deliver high charge in a little time and high catalytic activity. Besides, the developed sensor could detect Cr(VI) at a concentration level of 9.5 µg·L^−1^.

Recently, Jolanta Kochana et al. [117] reported a capacitive sensor for Cr(VI) determination. The proposed sensor based on the self-assembled monolayer (SAM) of functionalized thiol on the gold electrode surface. The SAM was prepared from the mixture of thiol-decanethiol and S-{12-[1-(pyridin-4-ylmethyl)-1H-1,2,3-triazol-4-yl]dodecyl}. The developed capacitive sensor displayed a detection limit of 1.612 × 10^−2^ µg·L^−1^.

## 3. Electrochemical Biosensors for Hexavalent Chromium Determination

As per definition of IUPAC, a biosensor is an integrated receptor-transducer device that combines biological sensing materials (bioreceptor) with a transducer. The interaction between the analyte and the biorecognition element is then converted to a measurable signal by means of the transduction system. Then, the signal is converted into a readout or display.

**Figure 4:**
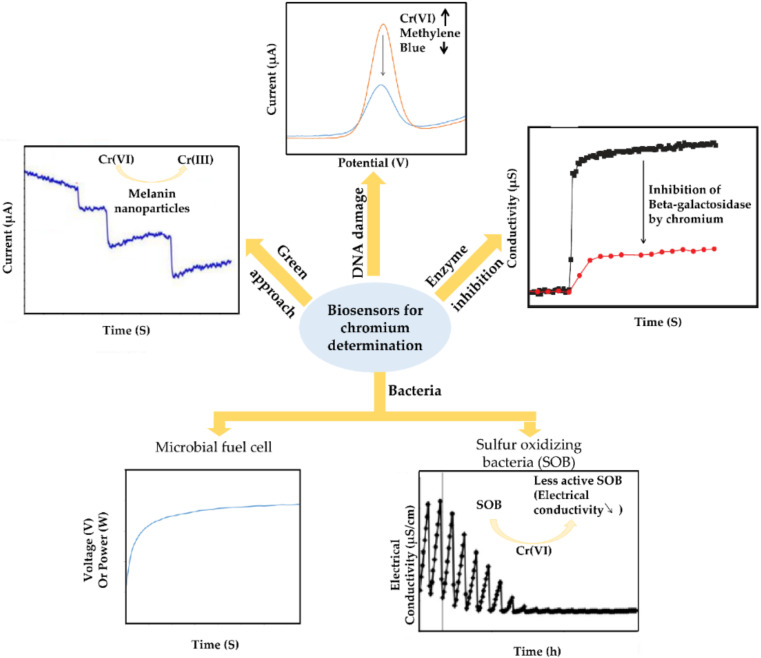
Scheme of different used biosensors for chromium determination.

To investigate the existence of chromium, many strategies are used for electrochemical biosensors modification (Figure 4). Microbial fuel cell (MFC) is one of the used approaches. These MFCs display four unique characteristics, no requirement neither of external enzymes nor the pure microorganism to be loaded, a rapid response of the anaerobic bacteria, no need of external power source owing to the MFCs’ capacity to generate electricity from wastewater, also the expectation of having different responses to several shocks incurred by the discharge of high concentrations of contaminants including heavy metals [22].

Zhiheng Xu et al. [118] reported a membrane based MFC. Carbon ink coated two compacting filter membranes intended for Cr(VI) monitoring. The proposed sensor provides high sensitivity and stability and also a high microporosity and hydrophilicity of membranes offering numerous advantages, among them, shortened acclimation duration (couple hours) as the microporosity minimizes the distance between cells leading to a faster acclimation and the hydrophilicity simplifies the process allowing a short time for incubation.

The development of MFCs with two chambers instead of one chamber has been effectuated by Hyeonyong Chung et al. [119] for Cr(VI) detection in water. The generated voltage was utilized to pursue the change in Cr(VI) concentration under different conditions in the co-presence of Fe(II) up to 1:15 molar ratio and in the co-presence of fulvic acid up to 50 mg·L^−1^. The developed warning device has successfully measured the Cr(VI) concentrations in the range from 100 to 1500 µg·L^−1^. Nevertheless, the proposed MFCs showed limitations in Cr(VI) detection at higher pH conditions. Which was overcame later [120,121]. Guey-Horng Wang et al. [120] suggested the use of an MFC based biosensor inoculated with a facultatively anaerobic, a Cr(VI)-reducing *Ochrobactrum anthropi* YC152 for Cr(VI) measurements in water. The *Ochrobactrum anthropi* YC152 displayed high adaptability to pH (5–8), salinity, temperature and quality of water under anaerobic conditions. Along with the increase of Cr(VI) concentration in the MFC, the MFC voltage decreases. The developed sensor showed two linear relationships between Cr(VI) concentration and voltage output were obtained 12.5–300 µg·L^−1^ and 300–5000 µg·L^−1^. Li-Chun Wu et al. [121] proposes an MFC Biosensor inoculated with Cr(VI)-reducing, *Exiguobacterium aestuarii* YC211, salt-tolerant, facultatively anaerobic and exoelectrogenic bacterium to evaluate its ability as a Cr(VI) biosensor. The proposed MFC biosensor was not affected by the parameters of the surrounding environment including the pH (5–9), temperature (20–35 °C), salinity and the coexisting ions. The MFC voltage output and the Cr(VI) concentration were inversely proportional in the range of 2500–60,000 µg·L^−1^.

A novel strategy based upon the integration of multi-anode microbial fuel cell (PMMFC) as a disposable self-support paper with a power management system (PMS) for real time Cr(VI) measurement in wastewater has been investigated by Zhiheng Xu et al. [122]. PMMFC sensor was tested at three types of shocks, Cr(VI) as the toxic metal, sodium acetate as the organic contaminant and sodium hypochlorous as the disinfectant in wastewater in a batch mode chamber. The developed PMMFC provides tremendous advantages including shorter acclimation duration (2–3 h) in comparison with traditional Carbon Cloth Anode MFC CCMMFC (3–15 days), enhanced power output four times than single anode MFC and six times than CCMMFC. The PMMFC demonstrates a higher sensitivity of power output compared to the voltage output [122].

Recently, Shuai Zhao et al. [123] explored a new Sediment Microbial Fuel Cell as a biosensor for real time and in situ monitoring of Cr(VI) in industrial wastewater. Under the optimized conditions (pH 6.4, temperature 25 °C) the developed biosensor displayed an excellent selectivity and accuracy, also a linearity ranges from 200 to 700 µg·L^−1^, also showed high specificity and no interference in the presence of other co-existing ions which indicate its high reliability.

As it was indicated in reference [22], the MFC based biosensor could be considered as a useful tool for Cr(VI) monitoring in wastewater as its voltage/power output are related to the activities of anaerobic electrogenic bacteria. The MFC with single chamber is limited by its long recovery time. Later, the MFC with two chambers was utilized for Cr(VI) measurement. However, its application was restricted in acidic medium (pH 1–2). Recently, PMMFC integrated PMS have utilized for Cr(VI) sensing along with sodium hypochlorous and sodium acetate. The PMMFC shortened the acclimation duration and enhanced the power output.

Aside from the microbial fuel cell sensors, the microbial biosensor can be determined as an analytical device which couples’ microorganisms (sulfur oxidizing bacteria (SOB), algae, nitrifying bacteria, iron oxidizing bacteria, bioluminescence bacteria and aerobic bacteria) with a physiochemical transducer to produce an electronic signal proportional to the analyte’s concentration. This type of sensors enables ease detection, cost effectiveness and good reproducibility. In the same context, Heonseop Eom et al. have established a sensing system based on the metabolic properties of SOB to oxidize sulfur (S^0^) and produce sulfuric acid (SO_4_^2−^) under aerobic conditions as it is shown in Equation (4) [22,124], leading to an augmentation in electrical conductivity (EC) and a lessening of pH.

S^0^ + H_2_O + 1.5O_2_ → SO_4_^2−^ + 2H^+^, ∆G^0^ = −587 kJ/reaction (4)

The presence of toxic substances leads to an inhibition in the SOB activity, causing less production of sulfuric acid, pH decreases and lower EC values. In their work, Heonseop Eom et al. [124] have elaborated a fed-batch bioreactor based on the use of SOB for measuring Cr(VI) in water. An effective concentration (EC_50_) of 1180 µg·L^−1^ was obtained after 2 h half exposure of Cr(VI) which makes it sensitive and suitable for the rapid determination of Cr(VI) in water.

Biosensors based upon enzyme inhibition were also used for Cr(VI) detection. In this type of biosensor, the analyte (Cr(VI)) inhibits the enzyme leading to a decrease of enzymatic product, which is related to the inhibitor (analyte) concentration. The principle of the enzymatic biosensor is based upon the quantification of the inhibitor, through the measurement of the enzymatic activity in both presence and absence of the inhibitor according to the following equation [125]: I(%) = [(A_0_-A_i_)/A_0_] × 100 where A_0_ is the activity in the absence of inhibitor and A_i_ is the activity in the presence of inhibitor. Using this approach, Fourou et al. [126] presented a selective, simple and sensitive enzymatic biosensor for toxic metals measurement including Cr(VI). The principle of the detection was based on the inhibition of β-galactosidase (β-gal) from *Aspergillus oryzoe* after its immobilization on an interdigitated gold electrode by cross-linking with glutaraldehyde. The enzyme inhibition by Cr(VI) was noted by the diminish in β-gal activity measured by the conductometric signal. Under the optimized conditions, the designed biosensor displayed a detection limit of 9.17 × 10^−2^ µg·L^−1^.

In another approach, Marta Jarczewska and coworkers [127] have proposed an alternative strategy for the determination of DNA damage caused by Cr(VI). In this approach, dsDNA has been adsorbed onto the gold disk electrodes surface by physical adsorption. The suggested assay banks on the measurement of the current change of methylene blue (MB) as an external redox indicator before and after its incubation in potassium chromate solution at pH 3. This method showed a more efficient manner of DNA-based assay preparation (ease of preparation, no preincubation in MB) in comparison with the already proposed studies that rely on the evaluation of DNA damage by MB redox active specie [128]. Furthermore, hydrogen peroxide and ascorbic acid were added to tune the degrading effect of CrO_4_^2−^. The developed biosensor operates effectively within an incubation time of 30 min with a linear range of 52–5200 μg·L^−1^.

A novel strategy based on the development of photoelectrochemical bioassay destined to Cr(VI) detection was proposed by Kheibar Dashtian et al. [129]. A photoactive lead sulfoiodide (Pb_5_S_2_I_6_) has been synthesized hydrothermally at a low temperature (160 °C), then, it was conjugated with the hydroxyl and amino groups of polydopamine followed by its coating on nanoporous TiO_2_ by in situ polymerization, which was grown on Ti foil by anodization. Ti/TiO_2_ electrode couples ideally with Pb_5_S_2_I_6_, achieving great photoelectrochemical properties. The fabricated sensor showed satisfactory results for Cr(VI) sensing with high stability, wide linear concentration ranges from 0.52 to 4160 µg·L^−1^ and a very low limit of detection of 0.156 µg·L^−1^. Such an advantageous sensor has provided high mobility of electrons and holes, conduction bands and mirror-imaged valence of the Pb_5_S_2_I_6_ high dielectric constant and its energy band matches that of TiO_2_ [130]. Furthermore, the Pb_5_S_2_I_6_ has a low rate of recombination of charge carriers which could be produced after light excitation (absorption of photons), conducting to a higher photo-to-electron out-put [131]. Moreover, the developed sensor provides an ease signal amplification for the sensitive bioassay of Cr(VI).

Recently, natural melanin nanoparticles (MNPs) have been utilized in biosensors elaboration as a green alternative to metal oxide and metallic nanoparticles [132]. Indeed, Gizem Kaleli-Can et al. [133] have described the elaboration of a new amperometric biosensor based upon the modification of a screen-printed carbon electrode (SPCE) by natural MNPs destined for Cr(VI) monitoring in water samples. SPCEs have been decorated by MNPs via layer-by-layer assembly (LBL-A) method for different cycle times and evaporation induced self-assembly technique (EI-SA). The incorporation of MNPs as biosensing material enhances the electrode response by 35 and 60 fold with the (LBL-A) and (EI-SA) technique respectively.

## 4. Electrochemical Sensors and Biosensors for Trivalent Chromium Determination

Oxidation state is a crucial factor usually overlooked in determining the toxicity of chromium species. Trivalent chromium which exists under several forms depending on the pH (Cr^3+^, CrOH^2+^, Cr(OH)_2_^+^, Cr(OH)_3_) is, in general, less toxic than the hexavalent chromium resulting in a few numbers of published papers dedicated to electrochemical sensors and biosensors for trivalent chromium monitoring.

Several nanocomposite sensors have been developed for Cr(III) monitoring [30,134,135,136]. In fact, ZAHRA HEIDARI et al. [134] reported a nanocomposite electrode based on MWCNT/Zeolite for Cr(III) sensing. Zeolite was used as a selective ionophore and MWCNTs for their attractive chemical and physical properties such as high chemical stability, good conductivity, good mechanical strength and maximum specific surface area [2]. An appropriate amount of each composite was mixed with carbon paste, then combined with potentiometry for Cr(III) measurement. The developed sensor displayed a stable current response toward Cr(III) in pH ranging from 3 to 10, which could be explained by no formation of hydroxyl complex and no protonation of zeolite. Also, it provides a fast response time (less than 10 s) in a linear range from 5.2 to 52 × 10^4^ µg·L^−1^ with a detection limit of 5.2 µg·L^−1^. Later, the same research group [135] reported a comparison between two modified carbon paste electrodes based on the use of zeolite for Cr(III) monitoring in different water samples with the recovery of 82–97%. Sensor 1 was modified by 3-Methylpyrazol-5-one and sensor 2 with chlorinated MWCNTs and they exhibit linear ranges of 52 to 52 × 10^4^ µg·L^−1^ and 5.2 to 52 × 10^4^ µg·L^−1^ respectively. The wider pH range, the wider linear range and the lowest detection limit were obtained by sensor 2 thanks to the presence of chlorinated MWCNTs that possibly increases the conductivity and improve the linear range, while shortest response time was obtained by sensor 1 which exhibits higher performance for Cr(III) detection than sensor 2 owing to the strong interaction between Cr(III) and 3-Methylpyrazol-5-one leading to smaller fluctuations in the potential difference. In another strategy where the glassy carbon electrode was modified by a nanocomposite of Chitosan and MWCNTs then used as a platform for immobilization of manganese oxide nanoflakes electrodeposited via a combination of cyclic voltammetry technique [30]. The fabricated sensor displays high electrocatalytic activity toward Cr(III) manganese oxide nanoflakes conduct to greatly enhanced electrochemical detection of Cr(III) owing to their reduced agglomeration and porous microstructure. Furthermore, the use of chitosan as a dispersant agent was advantageous for the electrochemical ability due to its high aptitude for the encapsulation of more manganese oxide. Under the optimal conditions, the fabricated sensor exhibits a stable response over the pH range of (3–7), high stability (3 months), good sensitivity, convenable reproducibility, minimal surface fouling, linear determination of Cr(VI) from 156 to 10,400 µg·L^−1^ with a convenient detection limit of 15.6 µg·L^−1^ [30]. Santhy Wyantuti et al. [137] reported the use of AuNPs modified GCE for Cr(III) measurement by differential pulse stripping voltammetry. The developed sensor showed the ability of Cr(III) monitoring in wastewater with a recovery of 97% at a very low concentration (0.01 µg·L^−1^).

Shixin Wu et al. [136] proposed the modification of the screen-printed electrode (SPE) by nanocomposite of chitosan-gold for Cr(III) monitoring in industrial wastewater with a great recovery of 99–100%. Amongst the prepared sensors including bare SPE, Au/SPE, Chi/SPE and Chi-Au/SPE. The Chi-Au/SPE provides the greatest oxidation response related to the Cr(III) thanks to the use of chitosan that offers a strong chelating ability toward Cr(III). Furthermore, the current response could be enhanced through Au incorporation leading to great electrochemical properties and high conductivity. Utilization of more than one nanostructure provides a lot of benefits in terms of sensitivity and selectivity owing to the synergistic effect between the different used nanomaterials. To avail of these benefits, Anish Khan et al. [138] reported a nanocomposite sensor based upon the modification of silver electrode by polymerization of aniline in presence of both reduced graphene oxide and tungsten oxide for Cr(III) monitoring at a concentration as low as 1.612 × 10^−^^3^ µg·L^−1^. The suggested sensor offers various advantages including low cost, short response-time of 10 s, good sensitivity, long term stability, wide linear range 5.2 × 10^−^^3^ µg·L^−1^ to 52 × 10^4^ µg·L^−1^ which are probably due to the surface porosity and high catalytic-decomposition activity of the nanocomposites.

Mohammad Musarraf Hussain et al. [139] have modified the glassy carbon electrode by a thin layer of (E)-N′-(4-Bromobenzyledene)-benzenesulfonohydrazide compound within the addition of nafion as polymer membrane (Nf/4-BBBSH/GCE). The addition of 4- BBBSH enhances the current intensity which could probably due to the formed complex between Cr(III) and 4-BBBSH. The fabricated sensor displays good selectivity toward Cr(III) determination in environmental samples and allows its detection at 4.97 × 10^−3^ µg·L^−^^1^ level.

Nanostructured ion imprinted polymer (IIP) on the platinum electrode was reported as a sensor modifier for Cr(III) monitoring in industrial wastewater with a recovery of about 97% [140]. The Cr(III)-IIP was prepared by using vinyl group functionalized MWCNTs, methacrylic acid, N, N’Methylene-bis-acrylamide, Cr (III) ion and potassium peroxo disulphate as the functional monomer, the cross linking agent, template molecule and initiator respectively. The use of MWCNT-IIP as a modifier enhances the electron transfer thanks to the adsorption of the analyte on its surface owing to the formed cavities by Cr(III). Moreover, the adsorption of Cr(III) was intensively influenced by the pH of the medium. It was greater at pH value less than 5, which could be explained by the formation of hydroxide at pH greater than 5 and protonation of the binding site at pH < 3. Differential pulse voltammetry was conducted for Cr(III) sensing with good sensitivity, selectivity and reproducibility.

A nanochannel biosensor platform was developed for Cr(III) and microRNA measurement by using a detachable molecular gate which is the nick hybridization chain reaction (nHCR) nanostructure, which responds to sequence Y. Transfer of Cr(III) into nucleic acid X has been carried out by Metal ion-dependent DNAzyme cleavage. The resulted nucleic acid X could be further amplified and converted into universal sequence Y. the disassembly of the nHCR nanostructure molecular gate turned on the ionic current signal inside the nanochannel, upon the addition of sequence Y into the nHCR functionalized nanochannel. The ON−OFF ratio showed a linearity with Cr(III) concentration ranges from 1.04 × 10^−5^ to 1.04 µg·L^−1^ [141].

Martin Leimbach et al. [142] reported the use of electrochemical quartz crystal microbalance (EQCM) for electrochemical characterization of deposited chromium from Cr(III) in solutions. The electroplating from the trivalent chromium solution was facilitated by the addition of benzoic sulfimide (BSI) which facilitates the reduction of Cr(III) to Cr(0).

The analytical performances of the cited modified sensors and biosensors for trivalent chromium measurement are summarized in Table 6.

## 5. Sensors and Biosensors-Based Chromium Speciation

To investigate the critical effects of chromium species on both ecological and biological lives, speciation analysis usually matters of interest. Chromium species exist under several forms depending on the pH value (Cr^3+^, CrOH^2+^, Cr(OH)_2_^+^, Cr(OH)_3_, HCrO_4_^−^, CrO_4_^2−^, H_2_CrO_4_, HCrO_4_^−^ and Cr_2_O_7_^2−^)) For that, different electrochemical sensors and biosensors have been developed to quantify the Cr(VI) or Cr(III) concentrations and total chromium through the oxidation of Cr(III) to Cr(VI) if the designed sensor is developed for Cr(VI) measurement or via reduction of Cr(VI) to Cr(III) if the developed sensor is intended to the determination of Cr(III).

In fact, as it was already mentioned Santhy Wyantuti et al. [143] have developed a sensor based on the modification of GCE by AuNPs for the detection of Cr(VI) and Cr(III). Cyclic voltammetry was conducted for the detection of both chromium species. However, their speciation was possible through the use of different mediums with different pH values. Oxidation of Cr(III) was carried out in acetate buffer at pH 5 and the reduction of Cr(VI) was effectuated in HCl (0.01M (pH = 2)).

In another study, a PEC sensor based upon the combination of Cr(VI) ion imprinted polymer and formate anion incorporated graphitic carbon nitride (IIP@F-g-C_3_N_4_) was developed and destined to Cr(VI) detection. The constructed sensor could also be used for total chromium quantification and that after the oxidation of Cr(III) by the use of hydrogen peroxide (H_2_O_2_) whereas Cr(III) content was calculated by subtracting the Cr(VI) concentration from total Cr [78]. Another research group has developed a specific sensor for chromium determination at ppt level [43]. The in situ electroplated GCE by bismuth film in the presence of a zinc mediator was combined with differential pulse cathodic adsorptive stripping voltammetry for Cr(VI) sensing. The determination of total chromium was accomplished after the oxidation of Cr(III) to Cr(VI) during UV-mineralization. Then, Cr(III) concentration was easily calculated by the subtraction of Cr(VI) from total chromium [43]. Wan Wang et al. [144] reported a disposable plastic electrode fabricated through the sputtering of a thin layer of gold on the surface of polyethylene terephthalate substrate. In this work, amperometry and adsorptive cathodic stripping voltammetry (AdCSV) techniques were conducted for Cr(VI) and total chromium measurements respectively. Total chromium detection was carried out after all chromium species reduction to Cr(III) by NH_2_OH·HCl followed by its complexation with 4-(2-pyridylazo) resorcinol (PAR). The formed complex PAR-Cr(III) was then measured by AdCSV. The elaborated method showed great advantages including its specificity, ease of detection, low cost and its reliability.

Screen-printed electrode (SPE) modified by chitosan–gold nanocomposites in combination with differential pulse stripping voltammetry (DPSV) was used for the measurement of Cr(III). However, total Cr monitoring was carried out after the addition of an excess of Na_2_SO_3_ which allows the reduction of all Cr(VI) to Cr(III). The use of chitosan-gold nanocomposite brings to a further improvement of the signal owing to the good conductivity of Au [136].

Subramanian Nellaiappan et al. [70] have developed a flow injection analysis (FIA) coupled with a dual electrochemical detector based on the modification of GCE by carbon nanofibers-chitosan decorated by gold nanoparticles as a sensing system for the speciation analysis of chromium. Chromium species detection was carried out by the same sensor in pH 2 PBS. The speciation ability of the prepared sensing system was confirmed by the use of a bipotentiostat, where an increase in the current response in FIA on electrode W_1_ for the oxidation of Cr(III) took place after Cr(III) addition, whereas no current response was observed on electrode W_2_ destined to the detection of Cr(VI) reduction and vice versa.

Electrochemiluminescence (ECL) sensor is a device based on the combination of visual luminescence and electrochemistry. As well as the potential applied on the electrode, its surface gets excited resulting in the production of electron transfer between molecules and the produced emitted light is measured. The studies reported in the literature with the ECL sensors for Cr(VI) sensing allow its speciation through its ability to quench the electrochemiluminescence signal [35,145]. Contrary to Cr(III), Cr(VI) showed the ability to quench the strong cathodic electrochemiluminescence signal of peroxodisulfate and graphene quantum dots in presence of EDTA which not the case with Cr(III) [35]. The same principle was reported by Hongmin Ma et al. with an ECL sensor based on gold nanoparticles-hybridized Pb (II)-β-cyclodextrin metal-organic framework [145].

Electroanalytical determination of Cr(VI) and Cr(III) was investigated using a new microbial biosensor based on the modification of carbon paste electrode (CPE) by a bacterial cell *Citrobacter freundii* [146]. The mechanism of detection is based on the binding of target ion onto the surface of bacterial cells followed by their reduction, wherein the preconcentration of each chromium species was carried out at different applied potential (+1V for Cr(VI) and −1V for Cr(III)). Moreover, the obtained reduction peaks depend on the pH of the medium and the oxidation state of the target (Cr(VI) or Cr(III)). The incorporation of the bacterial cell in CPE demonstrated an enhancement in performance (twofold) compared with bare CPE which can be explained by the exposure of functional groups in *Citrobacter freundii* towards the adsorption of ions [146]. Recently and in a similar context, Prabhakaran and coworkers [147] have elaborated a bacterial biosensor through the bio-modification of CPE by *Sphingopyxis macrogoltabida SUK2c* for Cr(VI) and Cr(III) monitoring in aqueous solution.

An evaluation of Cr(VI) and Cr(III) toxicities in water was carried out using sulfur-oxidizing bacteria (SOB) bioassays in batch and fed batch conditions [148]. The inhibition principle for SOB is the same as cited above by measuring electrical conductivity, pH and production of sulfate. Contrary to Cr(VI), it was demonstrated that Cr(III) has no toxicity for concentration up to 100 mg·L^−1^ in both conditions. At a concentration of 500, 1000 and 2000 µg·L^−1^ of Cr(VI), the activity of SOB was inhibited by 30, 60 and 98 percent respectively whereas at concentrations low than 100 µg·L^−1^, Cr(VI) did not affect the SOB activity.

Lanjunzi Liu [149] and coworkers have synthesized a biocomposite of polyNoradrenaline- horseradish peroxidase- glucose oxidase. Noradrenaline (NA) was used as the monomer and horseradish peroxidase (HRP) as the catalyst to carry out the polymerization of NA with simultaneous entrapment of glucose oxidase (GOx) and HRP. In this work, chromium speciation is based on the use of the described biocomposite in two different platforms. This biocomposite was casted on the platinum electrode for Cr(VI) determination. While with the addition of polyaniline (PANI), the resultant electrode was used for Cr(III) determination. The two mechanisms are based on enzyme inhibition where the inhibition percentage is proportional to the final inhibitor concentration in solution according to the aforementioned inhibition equation given. The prepared biosensor exhibited excellent performance in the determination of multiple analytes among them Cr(III) and Cr(VI) simultaneously with high sensitivity and selectivity. The fabricated PNA-HRP-GOx/Pt sensor showed an ability for Cr(VI) sensing at 1.04 × 10^−2^ µg·L^−1^ level with concentration ranges from 2.6 × 10^−2^ to 0.312 µg·L^−1^. PNA-HRP-GOx/PANI/Pt allows Cr(III) determination in the linear concentration range of 0.52–197.6 µg·L^−1^ [149].

## 6. Conclusions and Perspectives

Electrochemical methods for chromium analysis have intensively increased during the last five years, especially for hexavalent chromium. However, there are few studies concerning the determination of trivalent chromium due to its negligible toxicity comparing to hexavalent chromium.

This paper has summarized recent advances in both chromium species monitoring electrochemically. Compared to previous conventional/standard methods (Ion Chromatography, Atomic Absorption Spectrometry, Diphenylcarbazide colorimetric method) for chromium detection, the electrochemical techniques can be considered as a good alternative. They offer highly sensitive systems for chromium measurements with detection limits much lower than 2 µg·L^−1^ in most cases. The sensitivity and the conductivity of the electrochemical sensors could be enhanced alongside the increase of surface area through the incorporation of different sensing materials including carbon nanomaterials or metallic nanoparticles. A broad interest was given to the utilization of gold nanoparticles during the last five years due to their conductivity and large surface area. However, they sometimes showed limitations for hexavalent chromium detection in the presence of some co-existing ions such as chloride. Recently, in order to improve the sensitivity, increase the linear range, detect extremely low concentrations, an ultrasensitive sensor based on an electroplating of bismuth film with a reversible deposited zinc mediator was developed for hexavalent chromium determination. Similarly, the application of potential barrier for chromium monitoring can further develops the linear range and allows its determination at ppt level. Carbon black is used as the cheapest nanomaterial (1 euro/kg) [38] and is advantageously compared to the other nanomaterials for different applications.

Significant advances for electrochemical measurement of chromium were reported using nanocomposite sensors owing to their fascinating properties including the synergistic effect of the used nanomaterials, high surface area and, fast electron transfer rate. Recently, thanks to the attractive properties of the photoelectrochemical sensor, there is an increasing interest in this type of sensors for hexavalent chromium measurement with remarkable sensitivity, ease of integration and high selectivity of measuring chromium within the presence of different interfering species.

In addition, electrochemical biosensors are more and more utilized for chromium determination with a focus on hexavalent chromium owing to its critical effects on ecological and biological lives. Moreover, studies interested in speciation analysis of chromium species were reported based on pH of the medium, the activity of biological recognition element (generation of electricity, enzyme inhibition, electrical conductivity…) or by chemical or electrochemical modification of the sensor.

More optimizations of specificity, reusability, sensitivity and, stability are needed. Moreover, future research must enlarge the field of application by detecting chromium in other matrices than water samples. The on-line detection is highly required through the development of miniaturized devices for in situ and real-time monitoring.

## Figures and Tables

**Table 1:** Summary of the electroanalytical data of reported carbon nanomaterials based-electrodes for hexavalent chromium monitoring in several real samples.

Nano-Sensor	Modification Strategy	Technique	Linear Range (µg·L^−1^)	LOD (µg·L^−1^)	Real Sample	Reference
CNT/FTO	Coating by doctor blading	CV, amperometry	0–100	5	cooling tower blowdown water	[31]
CNT/flexible paper electrode	Direct painting on the Whatman cellulose filter paper					
CNT/SPE	Carbon printed					
Sol-gel/SWCNTs/GCE	electrodeposition	SWV	5–300	0.8	ashed swine blood	[32]
Graphene/GCE	Drop casting	DPCSV	10.4–52,000	7.8	Tap water	[34]
GQD/peroxo-disulfate system	chemical oxidation method	Electro-chemiluminescence	2.6–3120	1.04	river water	[35]
Phosphomolybdate based crystalline materials-CB/GCE	Drop casting	amperometry	26–19,656	1.35	Lake water	[39]
CB/GCE	Drop casting (15 µL)	amperometry	1.3–25,127.7	0.52	Not done	[40]

CB: carbon black; CNT: carbon nanotube; CV: cyclic voltammetry; DPCSV: differential pulse cathodic stripping voltammetry; FTO: fluorine tin oxide; GCE: glassy carbon electrode; GQDs: graphene quantum dots; LSV: linear sweep voltammetry; Pani: polyaniline; SPE: screen printed electrode; SWCNTs: single-walled carbon nanotubes; SWV: square wave voltammetry.

**Table 2:** Summary of the electroanalytical data of reported modified electrodes by metallic nanoparticles for hexavalent chromium monitoring.

Nano-Sensor	Modification Strategy	Technique	Linear Range (µg·L^−1^)	LOD (µg·L^−1^)	Real Sample	Reference
Bi/MCE	Spin coating- > Photolithography- > carbonization- > Etching and modification	AdSV	1–25	0.05	-	[42]
BiFE with zinc mediator/GCE	reduction of Bi(III) and Zn (II) to the metallic state at −1.1 V for 60 s Zn: reversible mediator	DPCAdSV	1.04 × 10^−5^–6.5 × 10^−5^	3.01 × 10^−6^	River Water	[43]
Ag plated-GCE	In situ plating	DP-ASV	18.2–2080	5.2	tap water	[45]
Ag NPs-BP-BPQ/GPE	Paste packed into the electrode	DPV	3 LR 4.16 × 10^−3^–0.52; 0.52–52; 52–5200	1.04 × 10^−4^	tap water, river water and electroplating wastewater	[46]
AuNPs/SPE	Drop casting 5µL	LSASV	0.7–35.0	1.6 × 10^−3^	tap and seawater	[47]
AuNPs/GCE	Self-assembly process	DPV	0.05–0.25	2.38 × 10^−3^	-	[49]
AuNPs/SPCE	Electrodeposition	LSV	20–200	5.4	River water	[50]
AuNS/CPSPE	Drop-casting	LSV	10–75	3.5	Contaminated groundwater	[51]
PET/nano-Au/Pt-RDE	Electrodeposition	DPAdCSV	2.5 × 10^−3^–40 × 10^−3^	10^−3^	Coastal water	[52]
AuNPs/CPE	AuNPs electrochemically deposited	DPV	40–3000	7	Tap water	[55]
AuNPs/CPE	AuNPs drop-casted		100–3000	54		
CPE			250–1500	26		
CPE in presence of DPC			50–260	19		

AdSV: adsorptive stripping voltammetry; Ag: silver; Ag NPs: silver nanoparticles; AuNPs: gold nanoparticles; AuNS: gold nanostars; Bi: bismuth; BiFE: bismuth film electrode; BP: biphenol; BPQ: biphenoquinone; CPSPE: carbon paste screen-printed electrode; CV: cyclic voltammetry; DPAdCSV: differential pulse adsorptive cathodic stripping voltammetry; DPASV: differential pulse anodic stripping voltammetry; DPCAdSV: differential pulse cathodic adsorptive stripping voltammetry; DPC: diphenylcarbazide; DPV: differential pulse voltammetry; GCE: glassy carbon electrode; GPE: graphite paste electrode; LSASV: linear sweep anodic stripping voltammetry, LSV: linear sweep voltammetry; MCE: mesoporous carbon electrode; PET: 4-pyridine-ethanethiol; Pt-RDE: platinum rotating disk electrode; SPCE: screen-printed carbon electrode; SPE: screen-printed electrode.

**Table 3:** Summary of the electroanalytical data of reported nanocomposite-based electrodes for hexavalent chromium monitoring.

Nanocomposite Sensor	Modification Strategy	Technique	Linear Range (µg·L^−1^)	LOD (µg·L^−1^)	Real Sample	Reference
Graphite/styrene-acrylonitrile copolymer composite electrode	Chemical method	SWASV	0–150	4.5	Tap water, river water, mineral water	[67]
IIP-S	One step electrodeposition	DPV	5.2 × 10^−2^–5.2	3.33 × 10^−2^	Tap water, river water	[68]
g-C_3_N_4_/AgM/Nf/GCE	Drop casting	amperometry	5.2–36.4	8.32 × 10^−2^	Tap, drinking, river and Industrial wastewater	[58]
NiFe NPs-CB/GCE	Drop casting	amperometry	1.3–5111.6	0.52	groundwater	[59]
3D NiO/PANI foam	Electrodeposition of PANI	DPV	0–235.3	2.06 × 10^−5^	-	[69]
AuNPs@CNF-CHIT/GCE	drop coating of 5 µL of CNF-CHIT and Au^3+^ followed by CV	Flow injection analysis coupled dual electrochemical detector (FIA-DECD)	100–100,000	0.32	wastewater	[70]
Au NPs/GR/GCE	Graphene/AuNPs (sonochemical method) Modification of GCE by drop-casting	amperometry	0–1040	0.52	River water	[60]
AuNPs/rGO/GCE	Electrodeposition by CV	SWV	5.2–1560	2.392	Lake water, river water	[71]
Ox.MWCNT-Au_nano_/Au	Drop casting 20 µL	amperometry	41.6–11,960	37.44	water	[61]
AuNPs/poly(aniline-co-o toluidine)/graphene oxide/AuE	Incubation of AuE in the copolymer (precipitation of a thin layer)	SWV	2600–26,000	1.118	Tap water	[62]
PANI/GQD-modified SPCE	electro-polymerization	LSV	100–10,000	97	Mineral water	[72]
Pyridine functionalized AuNPs/3D rGO/GCE	-3D RGO (electrochemical Reduction) -Electrodeposition of AuNPs -self-assembly of pyridine	AdSV	25–300	1.16	Wastewater (electroplate factories)	[73]
AuNPs/Nano-TiC/GCE	-Drop casting 10µL of Nano-TiC Electrodeposition of AuNPs	DPV	5.2–1040	2.08	coastal water	[74]
Ag-doped TiO_2_/ GCE	Drop casting	Amperometry	5–155	0.52	Tap water; lake water	[75]
Fe_3_O_4_/MoS_2_ /GCE	Drop casting	amperometry	52–136,760	26		[76]
AuPdNPs/ERGO/GCE	Electrodeposition by 2 ways (CV-amperometry)	DPV	2.6–260 and 260–52,000	0.676	Lake water, river water	[77]
Au-Pd nanoparticles/ITO	Electrodeposition by CV	LSV And chronoamperometry	0.052–5200	1.56 × 10^−2^	Tap and river	[66]

3D rGO: 3-dimensional reduced graphene oxide; AdSV: adsorptive stripping voltammetry; Ag: silver; AgM: silver molybdate; AuE: gold electrode; AuNPs: gold nanoparticles; AuPdNPs: bimetallic gold-palladium nanoparticles; CB: carbon black; CHIT: chitosan; CNF: carbon nanofibers; DPV: differential pulse voltammetry; ERGO: electrochemically reduced graphene oxide; Fe_3_O_4_: metallic nanoparticles; FIA-DECD: Flow injection analysis coupled dual electrochemical detector; GCE: glassy carbon electrode; g-C_3_N_4_: graphene carbon nitride; GQD: graphene quantum dot; GR: graphene; IIP-S: ion imprinted sensor; LSV: linear sweep voltammetry; MoS_2_: molybdenum disulfide; Nano-TiC: cubical nano-titanium carbide; Nf: nafion; NiFE: bimetallic NiFe nanoparticles; NiO: nickel oxide; Ox.MWCNT: functionalized multiwalled carbon nanotubes; Pani: polyaniline; rGO: reduced graphene oxide; SPCE: screen-printed carbon electrode; SWASV: square wave anodic stripping voltammetry; SWV: square wave voltammetry; TiO_2_: titanium dioxide.

**Table 4:** Summary of the electroanalytical data of reported photoelectrochemical sensors for hexavalent chromium monitoring.

Sensor	Modification Strategy	Technique	Linear Range (µg·L^−1^)	LOD (µg·L^−1^)	Real Sample	Reference
FTO (photoactive electrode)	Formate anion incorporated graphitic-carbon nitride (F-g-C_3_N_4_/IIP) Drop casting	Photoelectrochemical measurements	0.01–100.00	0.006	Water	[78]
Bi/BiOI-X/ITO (where x can be 1, 2, 3 or 4)	Drop casting (20µL)	photoelectrochemical detection	52–11,960	15.6	tap water, lake water	[81]
(BiPO_4_/BiOI/ITO)	Drop casting	Bi-SPR (Bismuth-surface plasmon resonance)	26–9360	7.8	Tap and lake water	[82]
MoS_2_/BiOI/ITO	Drop casting (20µL)	photoelectrochemical detection	2.6–520 520–8320	0.52	Tap and lake water	[83]
PbS QDs/ITO	Incubation Assembling with the linker poly(diallyl dimethyl ammonium chloride)	Photoelectrochemical detection	1.04 × 10^−3^–104	5.2 × 10^−4^	tap water, lake water	[80]
Au-TiO_2_/FTO	TiO_2_ nanorods: hydrothermal method AuNPs: electrodeposition by CV	chronoamperometry technique under chopped simulated solar light irradiation (100 mW·cm^−2^, light on/off cycles: 30 s)	0.52–2600	0.312	tap and river water	[79]
Au-TiO_2_/SPE	Screen printing of the TiO_2_ in the paste Electrodeposition of AuNPs by CV	Amperometry	0.52–5200	0.208	tap and river water	[84]
Au/TiO_2_ NPs/GCE	(Au@TiO_2_ NPs by hydrothermal method) Drop casting	amperometry	5200–140,400	520	Tap water, Industrial wastewater	[85]

Au: gold; Bi: bismuth; BiPO_4_: Bismuth phosphate; BiOI: Bismuth iodide oxide; GCE: glassy carbon electrode; FTO: fluorine tin oxide; ITO: indium tin oxide; MoS_2_: Molybdenum disulphide; PbS QDs: Lead(II) sulphide quantum dots; SPE: screen printed electrode; TiO_2_: titanium dioxide; TiO_2_ NPs: titanium dioxide nanoparticles.

**Table 5:** Summary of the electroanalytical data of reported modified gold solid electrodes for hexavalent chromium monitoring.

Nano-Sensor	Modification Strategy	Technique	Linear Range (µg·L^−1^)	LOD (µg·L^−1^)	Real Sample	Reference
MB/Au	Incubation	CV	26–52 × 10^4^	26	Not done	[114]
azacrown monolayer/Au	Self-assembly	EIS	1−100	0.0014	river water	[115]
Mn_3_O_4_/Nf/Au	Mn_3_O_4_ coating	CV	50–400	9.5	Canal water and Sewage water	[116]
Thiol monolayer/Au	Self-assembled monolayer	Capacitive measurement	0.052–0.52; 0.52–2.6; 5.2–26	1.612 × 10^−2^	synthetic solutions	[117]

Au: gold; CV: cyclic voltammetry; EIS: electrochemical impedance spectroscopy; Nf: nafion; MB: methylene blue.

**Table 6:** Summary of the electroanalytical data of reported electrochemical sensors and biosensors for trivalent chromium monitoring.

Nano-Sensor	Modification Strategy	Technique	Linear Range(µg·L^−1^)	LOD (µg·L^−1^)	Real Sample	Reference
MWCNT/zeolite/CPE	Incorporation in paste	Potentiometry	5.2–52 × 10^4^	5.2	Wastewater	[134]
Sensor 1: 3-Methylpyrazol-5-one/zeolite/CPE Sensor 2: chlorinated MWCNTs/zeolite/CPE	Incorporation in paste	potentiometry	52–52 × 10^4^ 5.2–52 × 10^4^	20.82.6	Drinking water, river water	[135]
MnOxNP/MWCNTs/Chit/GCE	Drop casting of MWCNs/ Chit. deposition of MnOxNP by CV	CV and amperometry	156–10,400	15.6	Drinking water	[30]
AuNPs/GCE	Self-assembly process	DPSV	0.5–75	0.01	wastewater	[137]
Chitosan-gold/SPE	electrodeposition	DPSV	52–5200	20.8	industrial wastewater	[136]
PANI-g-rGO@WO_3_/AgE	conducting binders: butyl carbitol acetate (BCA) and ethyl acetate (EA)	I-V	5.2 × 10^−3^–52 × 10^4^	1.612 × 10^−3^ ± 5.2 × 10^−4^	-	[138]
Nf/BBBSH/GCE	Drop casting	I-V	5.2 × 10^−3^–52 × 10^4^	4.97 × 10^−3^	Coal water, Industrial effluent, Red seawater, Tap water, Well water	[139]
MWCNT-IIP/Pt	Fabrication of paste of MWCNT-IIP on Pt electrode	DPV	1 × 10^3^–5 × 10^3^	266.1	industrial wastewater	[140]
Nanochannels with n-HCR		I-V	1.04 × 10^−5^–1.04	1.04 × 10^−5^	-	[141]
Quartz crystal electrode	Electroplating	EQCM	-	-	-	[142]

AgE: silver electrode; BBBSH: (E)-Nʹ-(4-Bromobenzylidene)-Benzenesulfonohydrazide; Chit: chitosan; CPE: carbon paste electrode; CV: cyclic voltammetry; DPSV: differential pulse stripping voltammetry; DPV: differential pulse voltammetry; EQCM: electrochemical quartz crystal microbalance; GCE: glassy carbon electrode; I-V: current-voltage; IIP: ion imprinted polymer; MnOxNP: manganese oxide nanoflakes; MWCNT: multiwalled carbon nanotubes; Nf: nafion; nHCR: nick hybridization chain reaction; PANI-g-rGO: polyaniline grafted graphene oxide; Pt: platinum; SPE: screen printed electrode; WO_3_: tungsten trioxide.

## References

[1] De Oliveira Farias E.A., dos Santos M.C., de Araujo Dionísio N., Quelemes P.V., Leite J.R.D.S.A., Eaton P., Eiras C. (2015). Layer-by-Layer films based on biopolymers extracted from red seaweeds and polyaniline for applications in electrochemical sensors of chromium VI. Mater. Sci. Eng. B.

[2] Arain M.B., Ali I., Yilmaz E., Soylak M. (2018). Nanomaterial’s based chromium speciation in environmental samples: A review. TrAC Trends Anal. Chem..

[3] Jin W., Yan K. (2015). Recent advances in electrochemical detection of toxic Cr(VI). RSC Adv..

[4] Gumpu M.B., Sethuraman S., Krishnan U.M., Rayappan J.B.B. (2015). Areview on detection of heavy metal ions in water-an electrochemical approach. Sens. Actuators B Chem..

[5] Aarthy M., Rajesh T., Thirunavoukkarasu M. (2020). Critical review on microbial fuel cells for concomitant reduction of hexavalent chromium and bioelectricity generation. J. Chem. Technol. Biotechnol..

[6] World Health Organization (2008). Guidelines for Drinking-Water Quality Third Edition Incorporating the First and Second Addenda Volume 1 Recommendations.

[7] Chromium in Drinking Water. https://www.epa.gov/sdwa/chromium-drinking-water#:~:text=EPA%20has%20a%20drinking%20water,to%20test%20for%20total%20chromium.

[8] Arancibia V., Nagles E., Gomez M., Rojas C. (2012). Speciation of Cr(VI) and Cr(III) in water samples by adsorptive stripping voltammetry in the presence of pyrogallol red applying a selective accumulation potential. Int. J. Electrochem. Sci..

[9] Grabarczyk M. (2008). Speciation analysis of chromium by adsorptive stripping voltammetry in tap and river water samples. Electroanalysis.

[10] Grabarczyk M., Kaczmarek L., Korolczuk M. (2007). Determination of Cr(VI) in the presence of complexing agents and humic substances by catalytic stripping voltammetry. Electroanalysis.

[11] Herrero-Latorre C., Barciela-García J., García-Martín S., Pena-Crecente R.M. (2018). Graphene and carbon nanotubes as solid phase extraction sorbents for the speciation of chromium: A review. Anal. Chim. Acta.

[12] Milačič R., Ščančar J. (2020). Cr speciation in foodstuffs, biological and environmental samples: Methodological approaches and analytical challenges—A critical review. TrAC Trends Anal. Chem..

[13] Özyol E., Saçmacı Ş., Saçmacı M., Ülgen A. (2018). A new turn-on fluorimetric method for the rapid speciation of Cr(III)/Cr(VI) species in tea samples with rhodamine-based fluorescent reagent. Spectrochim. Acta Part A Mol. Biomol. Spectrosc..

[14] López-García I., Marín-Hernández J.J., Hernández-Córdoba M. (2020). Speciation of chromium in waters using dispersive micro-solid phase extraction with magnetic ferrite and graphite furnace atomic absorption spectrometry. Sci. Rep..

[15] Sadeghi S., Moghaddam A.Z. (2014). Solid-phase extraction and HPLC-UV detection of Cr(III) and Cr(VI) using ionic liquid-functionalized silica as a hydrophobic sorbent. Anal. Methods.

[16] Aggrawal M., Rohrer J. (2016). Determination of Hexavalent Chromium Cr(VI) in Drinking Water by Suppressed Conductivity Detection I.

[17] Destanoğlu O., Gümüş Yılmaz G. (2016). Determination of cyanide, thiocyanate, cyanate, hexavalent chromium, and metal cyanide complexes in various mixtures by ion chromatography with conductivity detection. J. Liquid Chromatogr. Relat. Technol..

[18] Amin A.S., Kassem M.A. (2012). Chromium speciation in environmental samples using a solid phase spectrophotometric method. Spectrochim. Acta Part A Mol. Biomol. Spectrosc..

[19] Chang Q., Song S., Wang Y., Li J., Ma J. (2012). Application of graphene as a sorbent for preconcentration and determination of trace amounts of chromium (III) in water samples by flame atomic absorption spectrometry. Anal. Methods.

[20] He S., Lin X., Liang H., Xiao F., Li F., Liu C., Liu Y. (2019). Colorimetric detection of Cr(VI) using silver nanoparticles functionalized with PVP. Anal. Methods.

[21] American Public Health Association (1915). American Water Works Association, Water Pollution Control Federation, Water Environment Federation. Standard Methods for the Examination of Water and Wastewater.

[22] Biswas P., Karn A.K., Balasubramanian P., Kale P.G. (2017). Biosensor for detection of dissolved chromium in potable water: A review. Biosens. Bioelectron..

[23] Maduraiveeran G., Jin W. (2017). Nanomaterials based electrochemical sensor and biosensor platforms for environmental applications. Trends Environ. Anal. Chem..

[24] Maduraiveeran G., Sasidharan M., Ganesan V. (2018). Electrochemical sensor and biosensor platforms based on advanced nanomaterials for biological and biomedical applications. Biosens. Bioelectron..

[25] Zamora-Galvez A., Morales-Narváez E., Mayorga-Martinez C.C., Merkoçi A. (2017). Nanomaterials connected to antibodies and molecularly imprinted polymers as bio/receptors for bio/sensor applications. Appl. Mater. Today.

[26] Llobet E. (2013). Gas sensors using carbon nanomaterials: A review. Sens. Actuators B Chem..

[27] El Rhazi M., Majid S., Elbasri M., Salih F.E., Oularbi L., Lafdi K. (2018). Recent progress in nanocomposites based on conducting polymer: Application as electrochemical sensors. Int. Nano Lett..

[28] Amine A., Mohammadi H. (2018). Amperometry. Ref. Modul. Chem. Mol. Sci. Chem. Eng..

[29] Ghanam A., Lahcen A.A., Amine A. (2017). Electroanalytical determination of Bisphenol A: Investigation of electrode surface fouling using various carbon materials. J. Electroanal. Chem..

[30] Salimi A., Pourbahram B., Mansouri-Majd S., Hallaj R. (2015). Manganese oxide nanoflakes/multi-walled carbon nanotubes/chitosan nanocomposite modified glassy carbon electrode as a novel electrochemical sensor for chromium (III) detection. Electrochim. Acta.

[31] Wang C., Chan C.K. (2016). Carbon nanotube-based electrodes for detection of low ppb-level hexavalent chromium using amperometry. ECS J. Solid State Sci. Technol..

[32] Rosolina S.M., Bragg S.A., Ouyang R., Chambers J.Q., Xue Z.L. (2016). Highly sensitive detection of hexavalent chromium utilizing a sol-gel/carbon nanotube modified electrode. J. Electroanal. Chem..

[33] Carrington N.A., Yong L., Xue Z.L. (2006). Electrochemical deposition of sol–gel films for enhanced chromium (VI) determination in aqueous solutions. Anal. Chim. Acta.

[34] Liu C., He C., Xie T., Yang J. (2015). Reduction of graphite oxide using ammonia solution and detection Cr(VI) with graphene-modified electrode. Full-Nanotub. Carbon Nanostruct..

[35] Chen Y., Dong Y., Wu H., Chen C., Chi Y., Chen G. (2015). Electrochemiluminescence sensor for hexavalent chromium based on the graphene quantum dots/peroxodisulfate system. Electrochim. Acta.

[36] Cinti S., Arduini F., Carbone M., Sansone L., Cacciotti I., Moscone D., Palleschi G. (2015). Screen-Printed Electrodes Modified with Carbon Nanomaterials: A Comparison among Carbon Black, Carbon Nanotubes and Graphene. Electroanalysis.

[37] Arduini F., Di Nardo F., Amine A., Micheli L., Palleschi G., Moscone D. (2012). Carbon black-modified screen-printed electrodes as electroanalytical tools. Electroanalysis.

[38] Yammouri G., Mandli J., Mohammadi H., Amine A. (2017). Development of an electrochemical label-free biosensor for microRNA-125a detection using pencil graphite electrode modified with different carbon nanomaterials. J. Electroanal. Chem..

[39] Wang Y., Ma Y., Zhao Q., Hou L., Han Z. (2020). Polyoxometalate-based crystalline catalytic materials for efficient electrochemical detection of Cr (VI). Sens. Actuators B Chem..

[40] Liu D., Ji L., Ding Y., Weng X., Yang F., Zhang X. (2017). Mesoporous carbon black as a metal-free electrocatalyst for highly effective determination of chromium (VI). J. Electroanal. Chem..

[41] Chen S., Yuan R., Chai Y., Hu F. (2013). Electrochemical sensing of hydrogen peroxide using metal nanoparticles: A review. Microchim. Acta.

[42] Xu S., Wang X., Zhou C. A micro electrochemical sensor based on bismuth-modified mesoporous carbon for hexavalent chromium detection. Proceedings of the 2015 IEEE SENSORS.

[43] Tyszczuk-Rotko K., Madejska K., Domańska K. (2018). Ultrasensitive hexavalent chromium determination at bismuth film electrode prepared with mediator. Talanta.

[44] Oularbi L. (2018). Etude de Nanocomposites Polypyrrole/Nanoparticule de Carbone par Impédance Électrochimique et Ac-Électrogravimétrie: Application aux Capteurs Électrochimiques. Ph.D. Thesis.

[45] Stojanović Z., Koudelkova Z., Sedlackova E., Hynek D., Richtera L., Adam V. (2018). Determination of chromium (VI) by anodic stripping voltammetry using a silver-plated glassy carbon electrode. Anal. Methods.

[46] Shahbakhsh M., Noroozifar M. (2019). Ultra-trace determination of hexavalent chromium by novel two dimensional biphenol-biphenoquinone nanoribbons/silver nanoparticles. Sens. Actuators B Chem..

[47] Tukur S.A., Yusof N.A., Hajian R. (2015). Linear sweep anodic stripping voltammetry: Determination of Chromium (VI) using synthesized gold nanoparticles modified screen-printed electrode. J. Chem. Sci..

[48] Chirea M., Pereira C.M., Silva F. (2007). Catalytic effect of gold nanoparticles self-assembled in multilayered polyelectrolyte films. J. Phys. Chem. C.

[49] Wyantuti S., Ishmayana S., Hartati Y.W. (2015). Voltammetric determination of Cr (VI) using gold nanoparticles-modified glassy carbon electrode. Procedia Chem..

[50] Tu J., Gan Y., Liang T., Wan H., Wang P. (2018). A miniaturized electrochemical system for high sensitive determination of chromium (VI) by screen-printed carbon electrode with gold nanoparticles modification. Sens. Actuators B Chem..

[51] Dutta S., Strack G., Kurup P. (2019). Gold nanostar-based voltammetric sensor for chromium (VI). Microchim. Acta.

[52] Du Nguyen H., Nguyen T.T.L., Nguyen K.M., Tran T.A.T., Nguyen A.M., Nguyen Q.H. (2015). Determination of ppt level chromium (VI) using the gold nano-flakes electrodeposited on platinum rotating disk electrode and modified with 4-thiopyridinium. Am. J. Anal. Chem..

[53] Mohan D., Pittman C.U. (2006). Activated carbons and low cost adsorbents for remediation of tri-and hexavalent chromium from water. J. Hazard. Mater..

[54] Sankararamakrishnan N., Jaiswal M., Verma N. (2014). Composite nanofloral clusters of carbon nanotubes and activated alumina: An efficient sorbent for heavy metal removal. Chem. Eng. J..

[55] Hilali N., Ghanam A., Mohammadi H., Amine A., García-Guzmán J.J., Cubillana-Aguilera L., Palacios-Santander J.M. (2018). Comparison between modified and unmodified carbon paste electrodes for hexavalent chromium determination. Electroanalysis.

[56] Shrivastava S., Jadon N., Jain R. (2016). Next-generation polymer nanocomposite-based electrochemical sensors and biosensors: A review. TrAC Trends Anal. Chem..

[57] Shoaie N., Daneshpour M., Azimzadeh M., Mahshid S., Khoshfetrat S.M., Jahanpeyma F., Foruzandeh M. (2019). Electrochemical sensors and biosensors based on the use of polyaniline and its nanocomposites: A review on recent advances. Microchim. Acta.

[58] Karthika A., Nikhil S., Suganthi A., Rajarajan M. (2020). A facile sonochemical approach based on graphene carbon nitride doped silver molybdate immobilized nafion for selective and sensitive electrochemical detection of chromium (VI) in real sample. Adv. Powder Technol..

[59] Liu J., Ding Y., Ji L., Zhang X., Yang F., Wang J., Kang W. (2017). Highly sensitive detection of Cr (VI) in groundwater by bimetallic NiFe nanoparticles. Anal. Methods.

[60] Sari T.K., Takahashi F., Jin J., Zein R., Munaf E. (2018). Electrochemical determination of chromium (VI) in river water with gold nanoparticles–graphene nanocomposites modified electrodes. Anal. Sci..

[61] Breslin C.B., Branagan D., Garry L.M. (2019). Electrochemical detection of Cr (VI) with carbon nanotubes decorated with gold nanoparticles. J. Appl. Electrochem..

[62] Hussein M.A., Ganash A.A., Alqarni S.A. (2019). Electrochemical sensor-based gold nanoparticle/poly (aniline-co-o-toluidine)/graphene oxide nanocomposite modified electrode for hexavalent chromium detection: A real test sample. Polym. Plast. Technol. Mater..

[63] Li L., Chen X., Wu Y., Wang D., Peng Q., Zhou G., Li Y. (2013). Pd-Cu_2_O and Ag-Cu_2_O Hybrid Concave Nanomaterials for an Effective Synergistic Catalyst. Angew. Chem. Int. Ed..

[64] Wang D., Villa A., Porta F., Prati L., Su D. (2008). Bimetallic gold/palladium catalysts: Correlation between nanostructure and synergistic effects. J. Phys. Chem. C.

[65] Prati L., Villa A., Porta F., Wang D., Su D. (2007). Single-phase gold/palladium catalyst: The nature of synergistic effect. Catal. Today.

[66] Moakhar R.S., Hariri M.B., Kushwaha A., Dolati A., Ghorbani M., Goh G.K.L. (2016). Au-Pd bimetallic nanoparticle electrodes for direct electroreduction of hexavalent chromium complexes. Aust. J. Chem..

[67] Sari T.K., Jin J., Zein R., Munaf E. (2017). Anodic Stripping Voltammetry for the Determination of Trace Cr (VI) with Graphite/Styrene-Acrylonitrile Copolymer Composite Electrodes. Anal. Sci..

[68] Wu S., Dai X., Cheng T., Li S. (2018). Highly sensitive and selective ion-imprinted polymers based on one-step electrodeposition of chitosan-graphene nanocomposites for the determination of Cr (VI). Carbohydr. Polym..

[69] He Y., Zhao M., Yu M., Zhuang Y., Cheng F., Chen S. (2018). Interfacial potential barrier driven electrochemical detection of Cr^6+^. Anal. Chim. Acta.

[70] Nellaiappan S., Kumar A.S. (2018). A bipotentiostat based separation-free method for simultaneous flow injection analysis of chromium (III) and (VI) species. Electrochim. Acta..

[71] Liu Y., Gao G., Hu J., Zou X. (2018). Electrodeposited AuNPs/rGO nanocomposite as sensor for Cr (VI) determination in water. Int. J. Electrochem. Sci..

[72] Punrat E., Maksuk C., Chuanuwatanakul S., Wonsawat W., Chailapakul O. (2016). Polyaniline/graphene quantum dot-modified screen-printed carbon electrode for the rapid determination of Cr (VI) using stopped-flow analysis coupled with voltammetric technique. Talanta.

[73] Xu Y., Zhang W., Huang X., Shi J., Zou X., Li Z., Cui X. (2019). Adsorptive stripping voltammetry determination of hexavalent chromium by a pyridine functionalized gold nanoparticles/three-dimensional graphene electrode. Microchem. J..

[74] Han H., Pan D., Liu D., Hu X., Lin M., Li F. (2015). Cathodic stripping voltammetric determination of chromium in coastal waters on cubic nano-titanium carbide loaded gold nanoparticles modified electrode. Front. Mar. Sci..

[75] Ravishankar T.N., Muralikrishna S., Nagaraju G., Ramakrishnappa T. (2015). Electrochemical detection and photochemical detoxification of hexavalent chromium (Cr(vi)) by Ag doped TiO_2_ nanoparticles. Anal. Methods.

[76] Zhang Y., Chen P., Wen F., Yuan B., Wang H. (2016). Fe_3_O_4_ nanospheres on MoS_2_ nanoflake: Electrocatalysis and detection of Cr (VI) and nitrite. J. Electroanal. Chem..

[77] Hu J., Liu Y., Gao G., Zou X. (2018). One-step synthesis of AuPdNPs/ERGO nanocomposite for enhanced electrochemical sensing of Cr (VI) in water. J. Electrochem. Soc..

[78] Fang T., Yang X., Zhang L., Gong J. (2016). Ultrasensitive photoelectrochemical determination of chromium (VI) in water samples by ion-imprinted/formate anion-incorporated graphitic carbon nitride nanostructured hybrid. J. Hazard. Mater..

[79] Moakhar R.S., Goh G.K.L., Dolati A., Ghorbani M. (2017). Sunlight-driven photoelectrochemical sensor for direct determination of hexavalent chromium based on Au decorated rutile TiO_2_ nanorods. Appl. Catal. B Environ..

[80] Wang P., Cao L., Wu Y., Di J. (2018). A cathodic photoelectrochemical sensor for chromium (VI) based on the use of PbS quantum dot semiconductors on an ITO electrode. Microchim. Acta.

[81] Li M., He R., Wang S., Feng C., Wu H., Mei H. (2019). Visible light driven photoelectrochemical sensor for chromium (VI) by using BiOI microspheres decorated with metallic bismuth. Microchim. Acta.

[82] Li M., Zhang G., Feng C., Wu H., Mei H. (2020). Highly sensitive detection of chromium (VI) by photoelectrochemical sensor under visible light based on Bi SPR-promoted BiPO_4_/BiOI heterojunction. Sens. Actuators B Chem..

[83] Chen R., Tang R., Chen C. (2020). Photoelectrochemical detection of chromium (VI) using layered MoS 2 modified BiOI. J. Chem. Sci..

[84] Moakhar R.S., Goh G.K.L., Dolati A., Ghorbani M. (2015). A novel screen-printed TiO_2_ photoelectrochemical sensor for direct determination and reduction of hexavalent chromium. Electrochem. Commun..

[85] Ravishankar T.N., Vaz M.D.O., Ramakrishnappa T., Teixeira S.R., Dupont J. (2017). Ionic liquid assisted hydrothermal syntheses of Au doped TiO_2_ NPs for efficient visible-light photocatalytic hydrogen production from water, electrochemical detection and photochemical detoxification of hexavalent chromium (Cr^6+^). RSC Adv..

[86] Zheng Q., Vilà-Nadal L., Lang Z., Chen J.J., Long D.L., Mathieson J.S., Cronin L. (2018). Self-sorting of heteroanions in the assembly of cross-shaped polyoxometalate clusters. J. Am. Chem. Soc..

[87] Wang S.S., Yang G.Y. (2015). Recent advances in polyoxometalate-catalyzed reactions. Chem. Rev..

[88] Blazevic A., Rompel A. (2016). The Anderson–Evans polyoxometalate: From inorganic building blocks via hybrid organic–inorganic structures to tomorrows “Bio-POM”. Coord. Chem. Rev..

[89] Xin X., Ma Y., Hou L., Wang Y., Xue X., Lin J., Han Z. (2019). Krebs-Type {M_2_ (WO_2_)_2_ [B-β-SbW_9_O_33_]_2_}^n−^(M = Sb^III^,(WO_3_)) Tungstoantimonate Possessing Unique Pseudo-Seesaw Sb–O Structure. Inorg. Chem..

[90] Dong Y., Dong Z., Zhang Z., Liu Y., Cheng W., Miao H., Xu Y. (2017). POM constructed from super-sodalite cage with extra-large 24-membered channels: Effective sorbent for uranium adsorption. ACS Appl. Mater. Interfaces.

[91] Zhang Y., Liu J., Li S.L., Su Z.M., Lan Y.Q. (2019). Polyoxometalate-based materials for sustainable and clean energy conversion and storage. EnergyChem.

[92] Chen L., Chen W.L., Wang X.L., Li Y.G., Su Z.M., Wang E.B. (2019). Polyoxometalates in dye-sensitized solar cells. Chem. Soc. Rev..

[93] Du N., Gong L., Fan L., Yu K., Luo H., Pang S., Zhou B. (2019). Nanocomposites containing keggin anions anchored on pyrazine-based frameworks for use as supercapacitors and photocatalysts. ACS Appl. Nano Mater..

[94] Zang D., Huang Y., Li Q., Tang Y., Wei Y. (2019). Cu dendrites induced by the Anderson-type polyoxometalate NiMo_6_O_24_ as a promising electrocatalyst for enhanced hydrogen evolution. Appl. Catal. B Environ..

[95] Zhu D., Bai Z., Ma H., Tan L., Pang H., Wang X. (2020). High performance simultaneous detection of β-nicotinamide adenine dinucleotide and l-tryptophan in human serum based on a novel nanocomposite of ferroferric oxide-functionalized polyoxometalates. Sens. Actuators B Chem..

[96] Wang Q., Khungwa J., Li L., Liu Y., Wang X., Wang S. (2018). Fabrication of polyoxometalate/GO/PDDA hybrid nanocomposite modified electrode and electrocatalysis for nitrite ion, ascorbic acid and dopamine. J. Electroanal. Chem..

[97] Boussema F., Gross A.J., Hmida F., Ayed B., Majdoub H., Cosnier S., Holzinger M. (2018). Dawson-type polyoxometalate nanoclusters confined in a carbon nanotube matrix as efficient redox mediators for enzymatic glucose biofuel cell anodes and glucose biosensors. Biosens. Bioelectron..

[98] Xin X., Hu N., Ma Y., Wang Y., Hou L., Zhang H., Han Z. (2020). Polyoxometalate-based crystalline materials as a highly sensitive electrochemical sensor for detecting trace Cr(vi). Dalton Trans..

[99] Ferreira T.A., Rodríguez J.A., Galán-Vidal C.A., Castrillejo Y., Barrado E. (2018). Flow based determination of Cr (VI) by adsorptive cathodic stripping voltammetry on an immobilized magnetic poly (ionic liquid) modified electrode. Talanta.

[100] Fu L., Liu Z., Ge J., Guo M., Zhang H., Chen F., Yu A. (2019). (001) plan manipulation of α-Fe_2_O_3_ nanostructures for enhanced electrochemical Cr (VI) sensing. J. Electroanal. Chem..

[101] Wang W., Bai H., Li H., Lv Q., Zhang Q., Bao N. (2016). Carbon tape coated with gold film as stickers for bulk fabrication of disposable gold electrodes to detect Cr (VI). Sens. Actuators B Chem..

[102] Tan F., Cong L., Jiang X., Wang Y., Quan X., Chen J., Mulchandani A. (2017). Highly sensitive detection of Cr (VI) by reduced graphene oxide chemiresistor and 1, 4-dithiothreitol functionalized Au nanoparticles. Sens. Actuators B Chem..

[103] Izadyar A., Al-Amoody F., Arachchige D.R. (2016). Ion transfer stripping voltammetry to detect nanomolar concentrations of Cr (VI) in drinking water. J. Electroanal. Chem..

[104] Jaihindh D.P., Thirumalraj B., Chen S.M., Balasubramanian P., Fu Y.P. (2019). Facile synthesis of hierarchically nanostructured bismuth vanadate: An efficient photocatalyst for degradation and detection of hexavalent chromium. J. Hazard. Mater..

[105] Chen D.D., Yi X.H., Zhao C., Fu H., Wang P., Wang C.C. (2020). Polyaniline modified MIL-100 (Fe) for enhanced photocatalytic Cr (VI) reduction and tetracycline degradation under white light. Chemosphere.

[106] Low J., Jiang C., Cheng B., Wageh S., Al-Ghamdi A.A., Yu J. (2017). A review of direct Z-scheme photocatalysts. Small Methods.

[107] Zeleke M.A., Kuo D.H. (2019). Synthesis of oxy-sulfide based nanocomposite catalyst for visible light-driven reduction of Cr (VI). Environ. Res..

[108] Teixeira P.R.S., do Nascimento Marreiro A.S., de Oliveira Farias E.A., Dionisio N.A., Silva Filho E.C., Eiras C. (2015). Layer-by-layer hybrid films of phosphate cellulose and electroactive polymer as chromium (VI) sensors. J. Solid State Electrochem..

[109] Ferrigno R., Stroock A.D., Clark T.D., Mayer M., Whitesides G.M. (2002). Membraneless vanadium redox fuel cell using laminar flow. J. Am. Chem. Soc..

[110] Wang H.Y., Su J.Y. (2013). Membraneless microfluidic microbial fuel cell for rapid detection of electrochemical activity of microorganism. Bioresour. Technol..

[111] Li Z., Venkataraman A., Rosenbaum M.A., Angenent L.T. (2012). A Laminar-Flow Microfluidic Device for Quantitative Analysis of Microbial Electrochemical Activity. ChemSusChem.

[112] Ye D., Yang Y., Li J., Zhu X., Liao Q., Zhang B. (2015). A laminar flow microfluidic fuel cell for detection of hexavalent chromium concentration. Biomicrofluidics.

[113] Kowsalya B., Thampi V.A., Sivakumar V., Subramanian B. (2019). Electrochemical detection of Chromium (VI) using NiO nanoparticles. J. Mater. Sci. Mater. Electron..

[114] Korshoj L.E., Zaitouna A.J., Lai R.Y. (2015). Methylene blue-mediated electrocatalytic detection of hexavalent chromium. Anal. Chem..

[115] Wei J., Guo Z., Chen X., Han D.D., Wang X.K., Huang X.J. (2015). Ultrasensitive and ultraselective impedimetric detection of Cr (VI) using crown ethers as high-affinity targeting receptors. Anal. Chem..

[116] Bhanjana G., Rana P., Chaudhary G.R., Dilbaghi N., Kim K.H., Kumar S. (2019). Manganese oxide nanochips as a novel electrocatalyst for direct redox sensing of hexavalent chromium. Sci. Rep..

[117] Kochana J., Starzec K., Wieczorek M., Knihnicki P., Góra M., Rokicińska A., Kuśtrowski P. (2019). Study on self-assembled monolayer of functionalized thiol on gold electrode forming capacitive sensor for chromium (VI) determination. J. Solid State Electrochem..

[118] Xu Z., Liu B., Dong Q., Lei Y., Li Y., Ren J., Li B. (2015). Flat microliter membrane-based microbial fuel cell as “on-line sticker sensor” for self-supported in situ monitoring of wastewater shocks. Bioresour. Technol..

[119] Chung H., Ju W.J., Jho E.H., Nam K. (2016). Applicability of a submersible microbial fuel cell for Cr (VI) detection in water. Environ. Monit. Assess..

[120] Wang G.H., Cheng C.Y., Liu M.H., Chen T.Y., Hsieh M.C., Chung Y.C. (2016). Utility of Ochrobactrum anthropi YC152 in a microbial fuel cell as an early warning device for hexavalent chromium determination. Sensors.

[121] Wu L.C., Tsai T.H., Liu M.H., Kuo J.L., Chang Y.C., Chung Y.C. (2017). A green microbial fuel cell-based biosensor for in situ chromium (VI) measurement in electroplating wastewater. Sensors.

[122] Xu Z., Liu Y., Williams I., Li Y., Qian F., Zhang H., Li B. (2016). Disposable self-support paper-based multi-anode microbial fuel cell (PMMFC) integrated with power management system (PMS) as the real time “shock” biosensor for wastewater. Biosens. Bioelectron..

[123] Zhao S., Liu P., Niu Y., Chen Z., Khan A., Zhang P., Li X. (2018). A novel early warning system based on a sediment microbial fuel cell for in situ and real time hexavalent chromium detection in industrial wastewater. Sensors.

[124] Eom H., Hwang J.H., Hassan S.H., Joo J.H., Hur J.H., Chon K., Oh S.E. (2019). Rapid detection of heavy metal-induced toxicity in water using a fed-batch sulfur-oxidizing bacteria (SOB) bioreactor. J. Microbiol. Methods.

[125] Bachan Upadhyay L.S., Verma N. (2013). Enzyme inhibition based biosensors: A review. Anal. Lett..

[126] Fourou H., Zazoua A., Braiek M., Jaffrezic-Renault N. (2016). An enzyme biosensor based on beta-galactosidase inhibition for electrochemical detection of cadmium (II) and chromium (VI). Int. J. Environ. Anal. Chem..

[127] Jarczewska M., Ziółkowski R., Górski Ł., Malinowska E. (2015). Electrochemical Detection of Chromium (VI): Induced DNA Damage. J. Electrochem. Soc..

[128] Ensafi A.A., Amini M., Rezaei B. (2013). Detection of DNA damage induced by chromium/glutathione/H2O2 system at MWCNTs–poly (diallyldimethylammonium chloride) modified pencil graphite electrode using methylene blue as an electroactive probe. Sens. Actuators B Chem..

[129] Dashtian K., Ghaedi M., Hajati S. (2019). Photo-Sensitive Pb_5_S_2_I_6_ crystal incorporated polydopamine biointerface coated on nanoporous TiO_2_ as an efficient signal-on photoelectrochemical bioassay for ultrasensitive detection of Cr (VI) ions. Biosens. Bioelectron..

[130] Liu Y., Ma H., Zhang Y., Pang X., Fan D., Wu D., Wei Q. (2016). Visible light photoelectrochemical aptasensor for adenosine detection based on CdS/PPy/g-C3N4 nanocomposites. Biosens. Bioelectron..

[131] Wang G.L., Shu J.X., Dong Y.M., Wu X.M., Zhao W.W., Xu J.J., Chen H.Y. (2015). Using G-quadruplex/hemin to “switch-on” the cathodic photocurrent of p-type PbS quantum dots: Toward a versatile platform for photoelectrochemical aptasensing. Anal. Chem..

[132] Piacenti da Silva M., Fernandes J.C., de Figueiredo N.B., Congiu M., Mulato M., de Oliveira Graeff C.F. (2014). Melanin as an active layer in biosensors. Aip Adv..

[133] Kaleli-Can G., Ozlu B., Özgüzar H.F., Onal-Ulusoy B., Kabay G., Eom T., Mutlu M. (2020). Natural melanin nanoparticle-decorated screen-printed carbon electrode: Performance test for amperometric determination of hexavalent chromium as model trace. Electroanalysis.

[134] Heidari Z., Masrournia M., Khoshnood R.S. (2016). Fabrication a composite electrode based on MWCNT/Zeolite for potentiometric determination of Cr^3+^. Orient. J. Chem..

[135] Heidari Z., Masrournia M. (2018). A novel modified carbon paste electrode for the determination of chromium (III) in water. J. Anal. Chem..

[136] Wu S., Sekar N.C., Tan S.N., Xie H., Ng S.H. (2016). Determination of chromium (III) by differential pulse stripping voltammetry at a chitosan–gold nanocomposite modified screen printed electrode. Anal. Methods.

[137] Wyantuti S., Hartati Y.W., Firdaus M.L., Panatarani C., Tjokronegoro R. (2015). Fabrication of gold nanoparticles-modified glassy carbon electrode and its application for voltammetric detection of Cr(III). Int. J. Sci. Technol. Res..

[138] Khan A., Khan A.A.P., Rahman M.M., Asiri A.M., Alamry K.A. (2015). Preparation of polyaniline grafted graphene oxide–WO 3 nanocomposite and its application as a chromium (iii) chemi-sensor. RSC Adv..

[139] Hussain M.M., Asiri A.M., Arshad M.N., Rahman M.M. (2020). Synthesis, characterization, and crystal structure of (E)-N′-(4-Bromobenzylidene)-benzenesulfonohydrazide and its application as a sensor of chromium ion detection from environmental samples. J. Mol. Struct..

[140] Aravind A., Mathew B. (2018). Electrochemical sensor based on nanostructured ion imprinted polymer for the sensing and extraction of Cr (III) ions from industrial wastewater. Polym. Int..

[141] Zhu L., Miao M., Shao X., Du Z., Huang K., Luo Y., Xu W. (2019). A universal electrochemical biosensor using nick-HCR nanostructure as molecular gate of nanochannel for detecting Chromium (III) ions and microRNA. Anal. Chem..

[142] Leimbach M., Tschaar C., Schmidt U., Bund A. (2018). Electrochemical characterization of chromium deposition from trivalent solutions for decorative applications by EQCM and near-surface pH measurements. Electrochim. Acta.

[143] Wyantuti S., Hartati Y.W., Panatarani C., Tjokronegoro R. (2015). Cyclic voltammetric study of Chromium (VI) and Chromium (III) on the gold nanoparticles-modified glassy carbon electrode. Procedia Chem..

[144] Wang W., Bai H., Li H., Lv Q., Wang Z., Zhang Q. (2017). Disposable plastic electrode for electrochemical determination of total chromium and hexavalent chromium. J. Electroanal. Chem..

[145] Ma H., Li X., Yan T., Li Y., Liu H., Zhang Y., Wei Q. (2016). Electrogenerated Chemiluminescence Behavior of Au nanoparticles-hybridized Pb(II) metal-organic framework and its application in selective sensing hexavalent chromium. Sci. Rep..

[146] Prabhakaran D.C., Riotte J., Sivry Y., Subramanian S. (2017). Electroanalytical detection of Cr(VI) and Cr(III) ions using a novel microbial sensor. Electroanalysis.

[147] Prabhakaran D.C., Ramamurthy P.C., Sivry Y., Subramanian S. (2020). Electrochemical detection of Cr (VI) and Cr (III) ions present in aqueous solutions using bio-modified carbon paste electrode: A voltammetric study. Int. J. Environ. Anal. Chem..

[148] Qambrani N.A., Hwang J.H., Oh S.E. (2016). Comparison of chromium III and VI toxicities in water using sulfur-oxidizing bacterial bioassays. Chemosphere.

[149] Liu L., Chen C., Chen C., Kang X., Zhang H., Tao Y., Yao S. (2019). Poly (noradrenalin) based bi-enzyme biosensor for ultrasensitive multi-analyte determination. Talanta.

